# LRR Conservation Mapping to Predict Functional Sites within Protein Leucine-Rich Repeat Domains

**DOI:** 10.1371/journal.pone.0021614

**Published:** 2011-07-18

**Authors:** Laura Helft, Vignyan Reddy, Xiyang Chen, Teresa Koller, Luca Federici, Juan Fernández-Recio, Rishabh Gupta, Andrew Bent

**Affiliations:** 1 Program in Cellular and Molecular Biology, University of Wisconsin - Madison, Madison, Wisconsin, United States of America; 2 Department of Electrical and Computer Engineering, University of Wisconsin - Madison, Madison, Wisconsin, United States of America; 3 Department of Plant Pathology, University of Wisconsin - Madison, Madison, Wisconsin, United States of America; 4 Dipartimento di Scienze Biomediche, Università ‘G. d'Annunzio’, Chieti, Italy; 5 Life Sciences Department, Barcelona Supercomputing Center, Barcelona, Spain; Instituto de Biología Molecular y Celular de Plantas, Spain

## Abstract

Computational prediction of protein functional sites can be a critical first step for analysis of large or complex proteins. Contemporary methods often require several homologous sequences and/or a known protein structure, but these resources are not available for many proteins. Leucine-rich repeats (LRRs) are ligand interaction domains found in numerous proteins across all taxonomic kingdoms, including immune system receptors in plants and animals. We devised Repeat Conservation Mapping (RCM), a computational method that predicts functional sites of LRR domains. RCM utilizes two or more homologous sequences and a generic representation of the LRR structure to identify conserved or diversified patches of amino acids on the predicted surface of the LRR. RCM was validated using solved LRR+ligand structures from multiple taxa, identifying ligand interaction sites. RCM was then used for *de novo* dissection of two plant microbe-associated molecular pattern (MAMP) receptors, EF-TU RECEPTOR (EFR) and FLAGELLIN-SENSING 2 (FLS2). *In vivo* testing of *Arabidopsis thaliana* EFR and FLS2 receptors mutagenized at sites identified by RCM demonstrated previously unknown functional sites. The RCM predictions for EFR, FLS2 and a third plant LRR protein, PGIP, compared favorably to predictions from ODA (optimal docking area), Consurf, and PAML (positive selection) analyses, but RCM also made valid functional site predictions not available from these other bioinformatic approaches. RCM analyses can be conducted with any LRR-containing proteins at www.plantpath.wisc.edu/RCM, and the approach should be modifiable for use with other types of repeat protein domains.

## Introduction

The conservative nature of evolution causes selection of stable structures that nevertheless are modifiable for operation in varied processes. Proteins carrying repetitive domains such as leucine rich repeats (LRRs), ankyrin repeats or tetratricopeptide repeats are one common solution to these evolutionary demands [1]–[3]. A single class of repeat domain can interact with a wide array of chemically distinct ligands, yet each particular repeat protein shows high specificity for particular ligands. Consensus amino acid motifs have been identified for these repeat domains [1]–[5]. The consensus amino acids within a particular type of repeat provide a regular, stable scaffold to the domain, while non-consensus residues within the repeat allow variability in function [1]. The characteristic structure formed by a particular type of repetitive motif is identifiable by comparison of multiple solved protein structures, and can be used to predict the overall configuration of other protein domains sharing that repetitive motif. However, methods to query the variable portions of these repeat domains, to identify and understand the sites that control specialized functions, are less well developed.

LRR domains are a protein-ligand interaction domain found in many types of prokaryotic, eukaryotic, and viral proteins, including ubiquitin ligases, hormone receptors, enzyme inhibitors, and immune receptors in plants and animals [1], [6]–[12]. As annotated by Pfam, more than 500 different proteins encoded by the human genome contain LRRs, and there are over 1000 types of LRR-containing proteins in individual plants such as *Arabidopsis thaliana* or rice [4]. The ubiquity of this domain may be due to its ability to interact with a wide range of substrates including proteins, nucleic acids, lipids, and small molecule hormones. It is particularly compelling that in jawless vertebrate adaptive immune systems, antibodies are produced by shuffling hypervariable LRR repeats [13], [14]. The resultant receptors recognize a wide range of substrates with high affinity. Any single LRR domain can potentially interact with several different molecules, either simultaneously or asynchronously.

**Figure 1:**
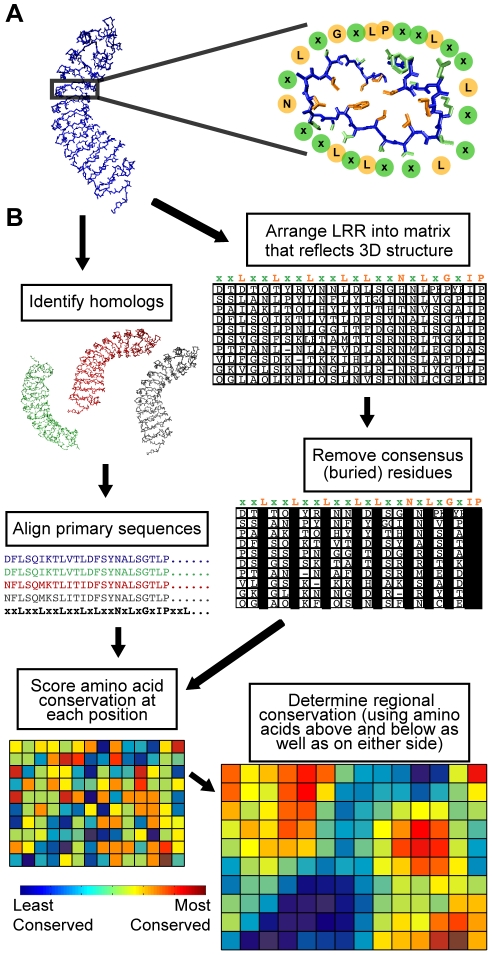
LRR structure and an outline of conservation mapping procedure. (**A**) Left: a representative LRR domain (left), *P. vulgaris* PGIP2, which forms the regular spiral pattern typical of LRRs. Right: a single 24 amino acid repeat of the LRR, surrounded by circles designating the residues of the LRR consensus amino acid sequence (xxLxxLxxLxxLxLxxNxLxGxIP). Note that the consensus residue side chains (orange) form the core of the protein, whereas the variable residues (green) are solvent-exposed. (**B**) Schematic representation of the conservation mapping procedure, using the example of PGIP1-4. See Methods and Text S1 for a detailed description of the procedure.

There are multiple sub-families of LRR domain types, each with slightly different consensus amino acid motifs, but all share the tendency to form a solenoid structure with approximately 20–30 amino acids per repeat where each repeat forms one turn of the helix (Figure 1A; an example of an LRR consensus motif is xxLxxLxxLxxLxLxxNxLxGxIP). The solenoid is curved so that a convex and a concave face are created. The concave face is largely composed of β-strands, and often forms the ligand binding site for LRR domains [15]. Deviations from the repeat consensus can result in intervening segments that interrupt adjacent solenoid regions, providing structural flexibility to the LRR. An LRR domain may be relatively brief (e.g. two or three repeats), or quite long (30 repeats or more). The consensus residues (mostly leucine or other hydrophobic amino acids) are usually buried in the core of the solenoid, while residues at the variable positions are predominantly solvent exposed (Figure 1A). The LRR structure efficiently creates a large surface-to-volume ratio protein domain that tolerates a wide variety of surface compositions, encoded in a condensed genomic space [16].

LRRs play a central role in the receptors that mediate two major branches of the plant immune system [17]. Many plant cell surface receptors for microbe- or pathogen-associated molecular pattern (MAMP/PAMP) molecules, which confer recognition of conserved microbial molecular motifs, contain LRRs as the bulk of their extracellular domain and a protein kinase intracellular domain [18]–[20]. The transmembrane LRR-kinase family of receptors is expansive in plants (over 200 different protein types in *Arabidopsis*, over 400 in rice [21]), and their functions extend well beyond immunity to include prominent roles in plant growth and development pathways [22], [23]. Plant MAMP receptors carry clear structural and functional analogies to (but apparent evolutionary independence from) animal MAMP receptors, the Toll-like receptors (TLRs), which also contain LRR domains [24]. LRRs are also central to recognition specificity in the large, diverse family of plant intracellular nucleotide binding (NB)-LRR proteins known as resistance (or “R”) proteins. These R proteins initiate strong defense responses upon recognition of specific pathogen effector molecules or upon recognition of a host protein alteration caused by a specific pathogen effector [11]. Plant R proteins are structurally similar to animal nucleotide-binding leucine-rich repeat (NLR) proteins that play significant recognition roles in the mammalian immune response [25]–[28].

Two plant LRR-kinase MAMP receptors that have been a particular focal point for research are ELONGATION FACTOR TU (EF-Tu) RECEPTOR (EFR) and the flagellin receptor FLAGELLIN-SENSING 2 (FLS2) [29]–[33]. FLS2 orthologs can be found in a wide range of monocotyledonous and dicotyledonous plant species, whereas EFR appears to be limited to the mustard family Brassicaceae. FLS2 can be activated by flagellin or by synthetic peptides that represent the highly conserved minimal recognition domain of flagellin, such as the 22 amino-acid flg22 peptide [29]. Similarly, EFR responds to conserved peptides from the recognized domain of bacterial EF-Tu, such as the elf18 peptide [32]. There is evidence that the LRR domains of these proteins directly interact with MAMP ligands [32], [34]–[36], but the large size of their LRR domains (22 repeats for EFR, 28 for FLS2) leaves open the possibility that these LRRs also mediate interaction with other ligands, co-receptor proteins and/or cofactors. FLS2, EFR and other MAMP receptors can be significant barriers to microbial infection [32], [37]–[42]. Intriguingly, transgenic tomato plants expressing *Arabidopsis* EFR are more resistant to the plant pathogenic bacteria *Ralstonia solanacearum* and *Xanthomonas campestris* pv. *vesicatoria*, implying that many of the mechanisms for downstream defense signaling from MAMP receptors are conserved across diverse plant species [38], [43].

Protein functional site prediction is already possible using a number of computational methods, each of which has certain advantages and limitations. Functional sites are often detectable as the sets of amino acids that have been most conserved or diversified among a set of homologous proteins [44]–[46]. Some computational methods require only primary sequences to look for conserved codons or amino acids within a sequence alignment, such as database searching for protein motifs (e.g., [47]) or alignment with homologous sequences (e.g., CLUSTAL). In contrast, positive/purifying selection analysis (e.g., Ka/Ks or dN/dS ratio; [46]) looks for amino acids that have undergone selection, either purifying or diversifying (positive), based on identification of non-neutral, selected substitutions at the nucleotide level among a group of homologous sequences. However, the above methods do not identify functional groupings of residues that are nearby in the folded protein but dispersed in the primary amino acid sequence. Some studies of positive selection on LRRs have manually approximated structural proximity to improve their power (e.g., [48]). Other computational approaches, such as Consurf [49], optimal docking area (ODA) [50], and conserved functional group (CFG) analysis [45], do use protein structural information obtained experimentally (X-ray or NMR) or by homology modeling. CFG and Consurf search homologous input sequences for conserved groupings of amino acids on the surface of the folded protein. ODA models desolvation energy, i.e. it searches for continuous surface patches that undergo favorable energy change when buried during a modeled protein-protein association; a low ODA value indicates a location that is predicted to interact with a ligand or another protein. These latter programs offer valuable paradigms but are limited to proteins with a crystal structure or a reliable homology model, and CFG and Consurf have constraints related to handling of hydrophobic and/or repeat motif residues that limit their efficacy for analysis of LRR domains.

In hundreds of important proteins, LRR domains contain the ligand specificity region or other functional sites, and there is significant interest in identification and manipulation of these sites. In the present study we developed the Repeat Conservation Mapping (RCM) program that predicts functional sites in LRR domains by identifying, among a group of homologous LRR-containing proteins, the patches of predicted spatially adjacent residues on the surface of the LRR that exhibit the greatest conservation or greatest divergence. The RCM method utilizes linear amino acid sequences as input, along with existing generalized LRR structure principles, to predict likely adjacency of residues in the LRR. It then identifies regional conservation scores for predicted surface residues based on conservation of that residue and its proximal surface amino acids. We validate the method using previously solved co-crystal structures for LRR with ligand, and through discovery and *in vivo* validation of previously unknown functional sites of the plant EFR and FLS2 MAMP receptors, for which structures are not currently available. The RCM program can be run using the publicly accessible web server at http://www.plantpath.wisc.edu/RCM, and the source code is openly available via a GNU general public license.

## Methods

### Repeat Conservation Mapping program

RCM analyses can be conducted at a publicly accessible web interface served from a Linux-based virtual machine. The RCM web site runs a set of linked php files that draw upon PHP, Perl, HTML, Python and C scripts, including local implementations of functions from ClustalW2 [51], [52], LRRScan [53] and MATLAB (The Mathworks, Inc., Natick, MA). The code is available under GNU general public license v3; local installation is not recommended for most end-users.

Figure 1B and the following briefly describe the method. The linear amino acid sequences of two or more LRR domains are aligned and compared, and a conservation score is determined for each amino acid position. Characteristic properties of LRR domains are then used to generate a generic super-helical structural model of the LRR domain being queried, which places amino acids in their likely relative locations to each other in a folded protein. A sliding window analysis then determines a center-weighted conservation score for all possible groups of adjacent amino acids that are predicted to reside on the surface of the helix. Typically a 5×5 matrix of 25 amino acids, spanning 5 LRR repeats, is queried to derive each regional conservation score. A colored heat-map of the result directs investigator attention to the most conserved (or most divergent) regions. Alternative score-weighting systems for the sliding window analysis are available to users; adjustment of these values significantly impacts the resulting conservation map. The default weighting values achieve a useful balance between the excessive smoothing (loss of resolution) that occurs with less center-weighting, and the loss of site identification (similarity to the initially calculated individual residue conservation scores) that occurs with greater center-weighting. Readers are referred to a more detailed step-by-step description of conservation mapping in Text S1.

### Comparisons to other methods

For positive selection analysis, sequence alignment and phylogenetic trees were constructed using MEGA 4.0 [54]; information from these trees were used to calculate positive selection using the CODEML module in PAML [46]. Optimal docking analysis (ODA) was implemented as described in [55]. The current web module of Consurf was used [49].

### Homology Modeling

The FLS2 homology model was obtained using the structure of PGIP2 from Phaseolus vulgaris as a template (PDB ID 1OGQ) [56]. Both proteins (as well as EFR) belong to the plant extracellular LRR protein subfamily [1] characterized by the same consensus sequence in the LRR domain, i.e. xxLxLxxNxLt/sGxIPxxLxxLxxL. Furthermore, the LRR domain is capped at the N-terminus and at the C-terminus by two small cysteine-rich domains, which are also evolutionarily conserved among PGIP and the RLK receptors FLS2 and EFR [57]. However, PGIP2 contains 10 LRRs matching the above consensus while FLS2 is characterized by 28 complete repeats, hence four separate alignments were manually prepared and used for homology modeling. The N-terminal and first 9 LRR repeats of PGIP2 were manually aligned to the N-terminal and first 9 LRR repeats of FLS2. The PGIP LRR domain was separately aligned to FLS2 repeats 7–15 and 13–21. A final alignment encompassed FLS2 repeats 20–28 plus the C-terminal flanking region. These alignments were used, together with the relevant PGIP2 coordinates, to obtain four independent partial models using Modeller, version 9.1 [58]. Twenty models were obtained from each alignment and the lowest energy models were selected according to the Modeller objective function. The four partial models obtained by this strategy are partially overlapping with two or three LRRs in common between consecutive ones. This allowed superposition of the models in their overlapping regions and merging of coordinates to obtain a single full-length model using the SSM algorithm as implemented in the program COOT [59]. This model was further energy minimized within Modeller to obtain a final model that encompasses residues 1 to 744 of FLS2. The geometrical quality of the model was very good as judged with PROCHECK [60] with 98.1% of residues lying in allowed regions of the Ramachandran plot, 1.2% in generously allowed regions and only 0.6% (4 residues) in the non allowed regions. The EFR LRR domain was aligned to FLS2 using ClustalW and the alignment was manually adjusted, when necessary, to match the plant extracellular LRR consensus sequence. An EFR homology model, encompassing residues 1–576, was calculated and energy minimized within Modeller using the FLS2 homology model as a template. The final model has a very good geometry with 96.8% residues in allowed regions of the Ramachandran plot, 2.4% in generously allowed regions and 0.8% (four residues) in non-allowed regions.

### EFR and FLS2 Constructs

The protein coding sequence of *Arabidopsis thaliana EFR* up to but not including the stop codon, along with native promoter sequence (1091 bp upstream of the start codon = 1074 bp upstream of the transcription start site), was amplified from Col-0 accession genomic DNA using the primers (CACCGGGTTTTTGTTTATTCAAAGATGGG and CATAGTATGCATGTCCGTATTTAACATCC) and cloned into pENTR/D-TOPO (Invitrogen). Mutations to *EFR* were made in this construct via PCR as in [35], using mutagenic primers and a high-fidelity DNA polymerase (typically Pfu Ultra II (Stratagene)), followed by DpnI treatment to digest template and transformation of the linear product into *E. coli*. Mutations were verified by DNA sequencing. Site-directed randomizing mutagenesis was performed as described in [35] using mutagenic ∼30 nt PCR primers in which only one codon was mutagenized using the degenerate codon NNB. Similarly, double-alanine mutants were created with a mutagenic primer of ∼30 nt with two selected codons mutated to alanine. To clone BrEFR1, primers (CACCATGAAGCCGTTTCTTTCAATTGCTCTACTCATG and CATTGTATGCATGTCCGCGCTTAACATCC) were designed to amplify the full-length best hit of *Arabidopsis* EFR, minus stop codon for fusion to C-terminal tag, for insertion into pENTR/D-TOPO. To clone *EFR* from other species, genomic DNA was extracted and the *EFR* LRR-encoding domain was amplified using primers based on the *EFR* sequences of *Arabidopsis* and *Brassica rapa*. Restriction enzyme sites (SbfI and FseI) flanking the LRR domain were engineered by site-directed mutagenesis into the *Arabidopsis EFR* promoter+coding region in the pENTR/D-TOPO vector. The *Arabidopsis EFR* LRR-encoding domain was then cut out of the vector. The LRR-encoding domains of the Brassicaceae *EFR* genes were amplified using species-specific primers with the restriction enzyme sites at the 5′-ends and cloned into TOPO vectors, then cut out of the TOPO vectors and ligated into the *Arabidopsis EFR* gene lacking the LRR domain. Sequences in pENTR/D-TOPO were moved, by LR Clonase II reaction (Invitrogen), into the Gateway vector pGWB13 (if using native EFR promoter) or pGWB14 (if using CaMV 35S promoter), fusing the EFR amino acid sequence to a C-terminal HA tag [61]. Constructs were then moved into *Agrobacerium tumefaciens GV3101* by electroporation and used to transform homozygous *Arabidopsis efr^−^* plants (SALK 068675c) using the floral dip method or for transient expression in *Nicotiana benthamiana* via *Agrobacterium* infiltration. Site-directed randomizing mutagenesis of FLS2 was as described in [35]. Constructs were transformed into homozygous *fls2-101* plants. All new DNA and derived amino acid sequences are deposited at Genbank under accession numbers JN002095-JN002103.

### Receptor function assays

Seedling growth inhibition assays were performed as described [35], with appropriate selection for transgenic seedlings prior to use. ROS assays were performed on leaf discs taken from 4- to 8-week old transgenic *Arabidopsis* or from 4- to 6-week old *N. benthamiana* leaves infiltrated two days prior with *A. tumefaciens* containing an *EFR*, *FLS2* or corresponding empty vector construct. Leaf discs were floated on 1% DMSO overnight and then treated with 1 µM peptide (or no peptide in the case of mock) in the presence of 1 µg/mL luminol and 1 µg/mL horseradish peroxidase. Luminescence was measured on a Synergy HT Microplate Reader (Bio-Tek) for 30 minutes following addition of peptide [32], [62], [63]. For callose deposition assays, seedlings were grown for 5 days on 0.5× MS agar and then transferred to liquid 0.5× MS (500 µl per well in 24-well plate) with elf18 or flg22 peptide at the indicated concentrations. After 24 hours in liquid, seedlings were fixed with 2% formaldehyde/5% acetic acid/60% ethanol (FAA), cleared overnight in 95% ethanol, stained with 0.01% aniline blue and viewed under an epifluorescence microscope to visualize callose deposits [62], [63]. For receptor protein detection, six to eight 3-week old seedlings were ground in 2× SDS buffer (2 mL buffer per g tissue), boiled, and centrifuged to remove particulates. 50 µl per sample was separated by SDS-PAGE, blotted onto a PVDF membrane, and detected using an anti-HA antibody conjugated to horseradish peroxidase (Roche), made visible using the ECL Plus Kit (Amersham).

## Results

### Rationale and description of LRR conservation mapping approach

LRRs have a regular structure in which a single repeat forms one turn within the overall super-helical structure. Across numerous solved LRR structures, the LRR consensus residues form the buried core of this configuration (e.g., Figure 1A) [1]. Because of the regularity of these LRR structures, two assumptions can be made for conservation mapping: 1) consensus residues are not on the protein surface; and 2) the repetitive structure of LRRs allows prediction of relative amino acid positions in the tertiary structure without requiring a crystal structure or a detailed homology model of the protein in question. Assumption 1 allows elimination of the highly conserved but functionally less revealing consensus residues from the analysis; assumption 2 allows prediction and assessment of spatially adjacent groups of surface residues that are not adjacent in the primary amino acid sequence. Following this rationale, we devised and implemented Repeat Conservation Mapping (RCM), a set of algorithms to identify predicted functional sites of LRR domains. RCM accomplishes this by identifying the extent of conservation of different amino acid patches on the predicted surface of LRR domains (see also program description in Text S1).

As an example, the lower-right element of Figure 1B shows RCM output for an extensively studied plant LRR domain-containing protein, POLYGALACTURONASE INHIBITOR PROTEIN (PGIP). PGIP was the first LRR-containing plant protein to have its structure solved [56]. In bean (*Phaseolis vulgaris*), there are four PGIPs, designated PGIP1-4, which have varied but overlapping specificities for different polygalacturonases (PGs) [64]. The RCM map for these four paralogs highlights several divergent and conserved patches (Figure 1B). Interestingly, the three positions in PvPGIP2 that, when mutated to alanine, have a significant negative impact on inhibitor activity [55], are all located in divergent patches identified by conservation mapping, perhaps indicating that these residues are also responsible for differences in receptor specificity. Indeed, a single amino acid that can switch specificity between PGIP1 and PGIP2 is also found in this divergent region [65].

### Validation: Conservation mapping highlights functionally significant regions of LRR domains

RCM highlights, among a group of homologous proteins, regions that are highly conserved or highly divergent on the surface of the LRR domain. To verify that RCM identifies significant functional sites, we utilized RCM to analyze all proteins in PDB that, as of October 2010, had a structure for an LRR domain interacting with another protein or a ligand, and for which at least one functional homolog could be identified. Two examples, RIBONUCLEASE INHIBITOR (RI), and TRANSPORT INHIBITOR-RESPONSIVE 1 (TIR1) are discussed here; the maps and analyses of the other nine protein groups are provided as Figure S1. For all of these RCM analyses, proteins were compared to at least three high-scoring homologs identified through BLAST and/or literature searches. An amino acid was considered to be interacting with ligand if identified in the article(s) accompanying the crystal structure(s) [66]–[76].

**Figure 2:**
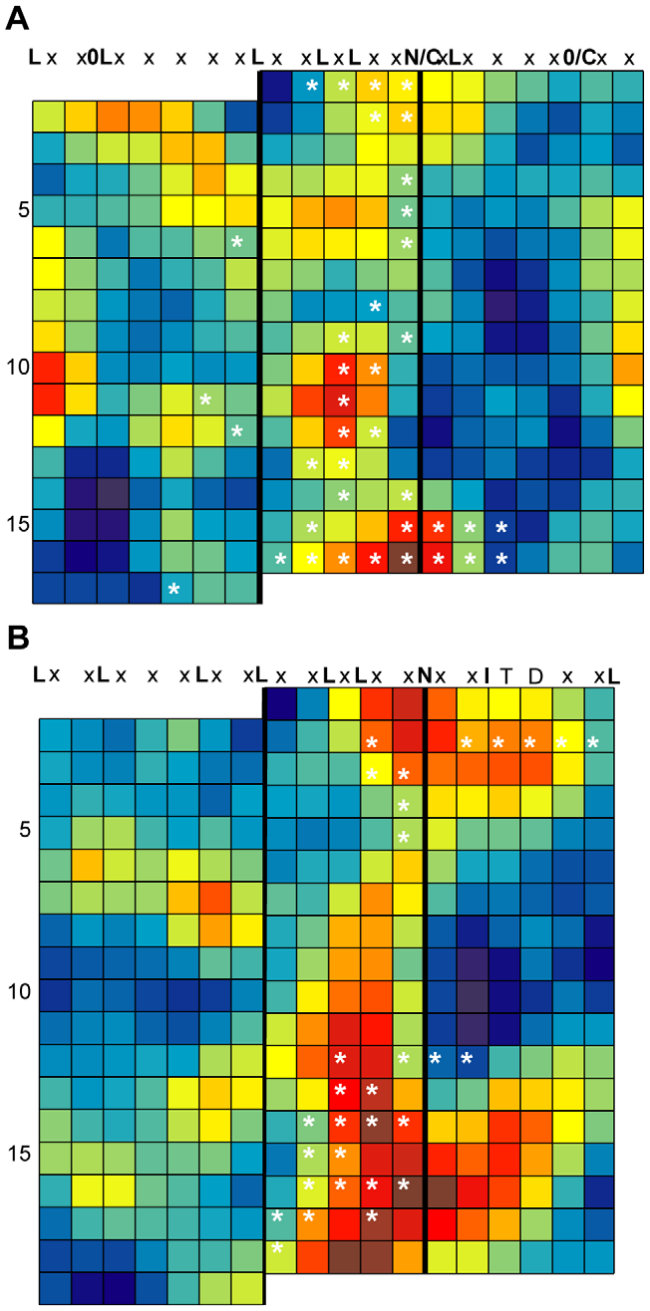
Validation of RCM by mapping LRR domains for which there are solved crystal structures with ligand. For all maps, each row represents a single repeat of the LRR, with each colored box representing a solvent exposed (non-consensus) amino acid position. Each column corresponds to a position within the LRR consensus sequence, as denoted at the top of each map. The color in each box reports the center-weighted regional conservation score for the 5×5 set of boxes that centers on that box (see text for details); dark red indicates the most conserved regions and blue indicates the most divergent regions (see scale bar in Fig. 1). Bold black vertical lines delineate the five residues in each row that comprise the β-strand, β-turn region (the convex face of the LRR domain). White asterisks were added after RCM and indicate amino acid positions that are LRR-ligand contact points in solved crystal structures. (**A**) RCM output for Ribonuclease inhibitor (RI) from human, rat, mouse and pig ribonuclease inhibitor (RI) LRRs. (**B**) RCM output for TIR1 and AFB1-5 (auxin receptors) from *Arabidopsis*.

RI is one of the most extensively studied LRR-containing proteins, with many reported crystal structures and mutagenesis studies (for a review, see [77]). RI prevents ribonucleases (RNases) from acting by binding to their catalytic domains with femtomolar affinities. There are two solved structures of RI bound to ribonucleases, human RI complexed with angiogenin, and porcine RI complexed with RNaseA. Binding of angiogenin also induced homodimerization of RI. These two RIs, along with RI from rat and mouse, were used to generate the conservation map in Figure 2A. Residues involved in any of the three interactions (hRI-angiogenin, sRI-RNAse A, hRI-hRI) are highlighted in Figure 2A with asterisks. Although many ligand contact sites could be seen in the crystal structure, kinetic analyes of mutants in these regions demonstrated that mutation of the locations identified by RCM as most conserved, that is, the residues in the β-strand, β-turn region of LRRs 10, 12, and 14–16, have the largest impact on ligand binding [77].

TIR1 is an auxin receptor in *Arabidopsis* and other plants; it ubiquitinates target proteins when it binds auxin hormones in a pocket formed by the LRR domain of the protein and the cofactor inositol-6 phosphate (InsP_6_). To investigate recognition of auxin by these receptors, we constructed a conservation map of *Arabidopsis* TIR1 with its five paralogs. In this case, the five *Arabidopsis* paralogs (AUXIN SIGNALNG F-BOX (AFB) 1–5) have all been implicated in auxin signaling [78]–[81]. Again, RCM successfully highlighted two patches on the surface of the LRR (Figure 2B): one where TIR1 binds auxin, and a second site where TIR1 binds auxin as well as the cofactor InsP_6_ [68]. For comparison, it can be valuable to see the individual residue conservation scores for the RI and TIR1 protein sets (Figure S2), which are obtained after alignment of primary amino acid sequences, but before sliding-window calculation of regional conservation scores that are shown in Figure 2. Visual inspection of the RCM maps for the other validation proteins (Figure S1) again indicates successful identification by RCM of sites involved in LRR+ligand interactions, in follicle stimulating hormone receptor, glycoprotein 1b alpha, Skp2, Slit, and TLR3. Poor success was obtained for TLR1, TLR2, TLR4 and TLR6. However, the ligand specificity is not known for many of the homolog sequences that were used, and they may not have been appropriate proteins to compare via RCM (see Discussion).

**Figure 3:**
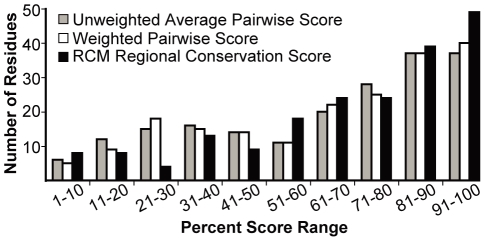
Residues involved in intramolecular interactions receive high scores in conservation mapping. For each map in Figures 2 and S1 (eleven protein families), all scores were arranged in descending order and then divided into deciles. Then, across all maps, the number of occurrences of residue positions marked by asterisks (LRR+ligand contact points) were tallied for each decile. Histogram shows the frequency with which three different types of scores fell into a particular decile. Score types are raw average pairwise BLOSUM65 score (grey), weighted average pairwise BLOSUM65 scores (see “Step 4” in Methods) prior to adjustment based on nearby amino acids (white), and regional conservation score that serves as final RCM output (black). Note that a subset of these protein comparisons involved homologs with unclear and possibly divergent rather than fully overlapping functions, in which case LRR-ligand contact points might be predicted to score as divergent rather than as conserved (see text).

Continuing with the above validation, combined data were analyzed for all conservation maps generated for the above proteins with known LRR+ligand structures. The regional conservation scores for residue positions known to be involved in LRR+ligand interactions were significantly higher than the set of all other residue scores in each RCM map generated (Student's T-test, p-value 0.005 or less). We also ranked the scores for each map into deciles and then determined the distribution of regional conservation scores, and individual residue conservation scores, for known LRR+ligand contact positions. Despite the presence of many potentially misleading TLR comparisons in the dataset, the distribution of scores for LRR+ligand interaction residues is weighted towards the highest ten percent of the RCM scores on their respective maps (Figure 3). Importantly, the proportion of scores in this decile increases when weighted pairwise comparisons are utilized (see Step 4 in Methods), and the proportion increases further when regional conservation scores are used (from 18% to 20% to 25% of interacting residues, respectively; Figure 3). 45% of all interacting residues appeared in the top 20% of RCM scores in this analysis. The remaining high-scoring sites identified by RCM may be involved in other functional processes that are not detected in the available LRR+ligand crystal structures, such as interaction with other ligands, cofactors or co-receptor proteins (see Discussion). Comparison of homologous proteins with diversified functions is also addressed below.

### Mutations of AtEFR at conserved locations identified by RCM often disrupt receptor function

RCM was used as a *de novo* discovery tool in experiments on EFR, the EF-Tu receptor of plants, for which there is no known structure or ligand binding site. To identify an EFR homolog for comparison, the derived amino acid sequence of *Arabidopsis* EFR was used with BLASTP to query all publicly available sequences from the Brassica Genome Gateway as of April 2009, and two high-scoring matches from *Brassica rapa* were identified. One of these sequences was cloned and then transiently expressed in *Nicotiana benthamiana* leaf mesophyll tissues, which do not otherwise respond to the EF-Tu-based elf18 peptide [32]. The defense mechanisms initiated by EFR or FLS2 include production of reactive oxygen species, release of other directly antimicrobial compounds, and production of callose and lignin as part of a cell wall strengthening response that limits pathogen penetration [82]. Plant seedlings undergoing chronic defense activation display inhibition of growth after multiple days of exposure to a recognized MAMP; this is a sensitive and widely used assay for FLS2 or EFR activation [31], [32]. The cloned *Brassica rapa EFR* homolog, designated Br*EFR1*, conferred recognition of 1 uM elf18 in an ROS assay (Figure S3). Br*EFR1* subsequently was shown to complement an *Arabidopsis efr^−^* mutant, rescuing the ability of the plants to respond to elf18 in ROS and seedling growth inhibition assays (Figure S3 and data not shown).

**Figure 4:**
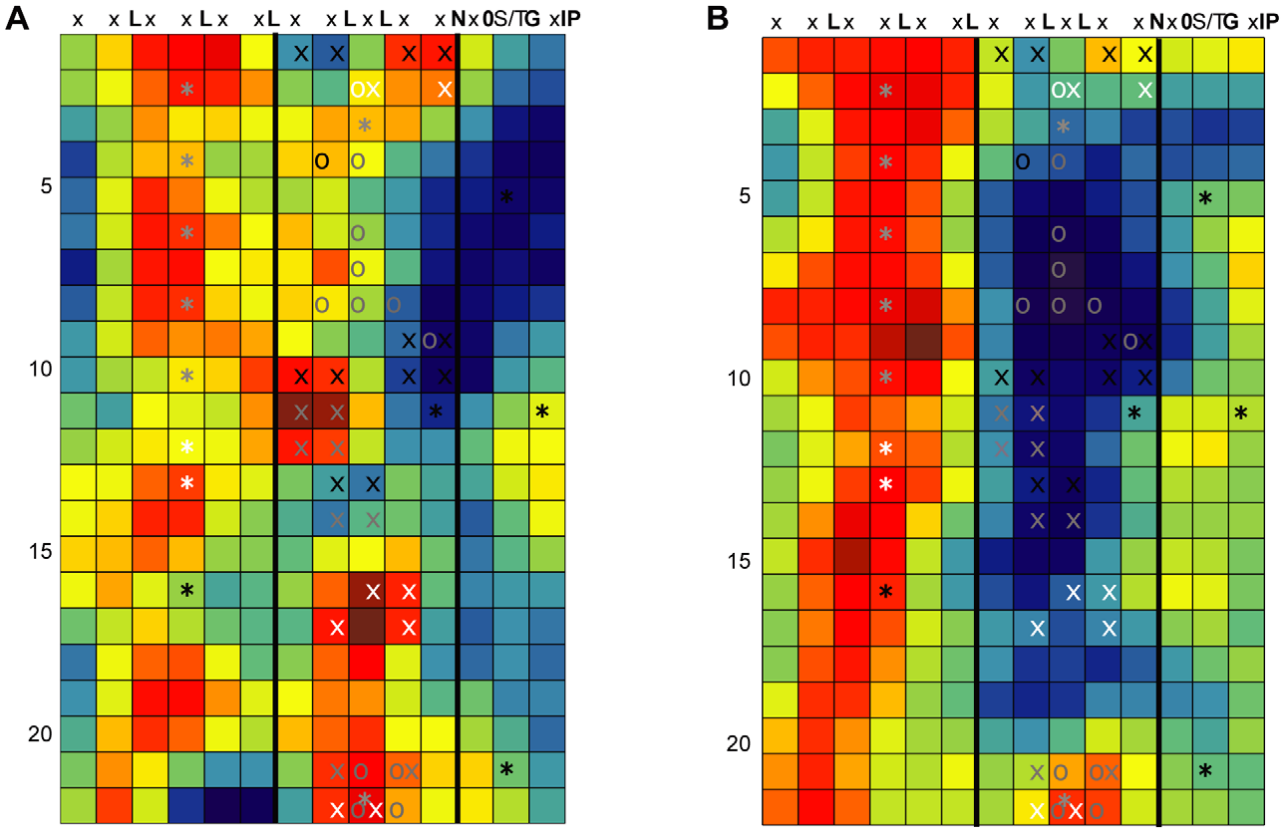
Conservation mapping of EFR. RCM maps of LRR domains (as described in Figure 2) depicting: (**A**) Conservation of AtEFR and a *Brassica rapa* homolog shown to have EFR activity. (**B**) Conservation mapping of AtEFR and its four most closely related *Arabidopsis* EFR paralogs, which fail to confer elf18 recognition in an *Arabidopsis efr^−^* mutant. The pairs of X symbols mark sites of double-alanine mutations, and O symbols mark sites of site-directed randomizing mutagenesis. The asterisks mark predicted sites of N-glycosylation as reported in [89]. Both (A) and (B) show the same set of X, O and * symbols. Based on data from Figures 5 and 6 and [89], symbols are white if the mutation disrupts EFR function, black if it does not, and grey for a partial/intermediate impact (less responsive than 95% of positive controls transformed with wild-type *EFR* but more responsive than 95% of empty-vector *efr^−^* negative controls; or statistically different from both wild-type and empty vector controls).

RCM was used to create a conservation map of AtEFR with BrEFR1 (Figure 4A). The map highlights several small patches of conservation on the surface of EFR, the highest scoring (most conserved) of which appear on the concave face of the receptor. The convex face also contains patches of conservation (the third and fourth columns of Figure 4A). There is a large patch of divergence in LRRs 3 through 10.

**Figure 5:**
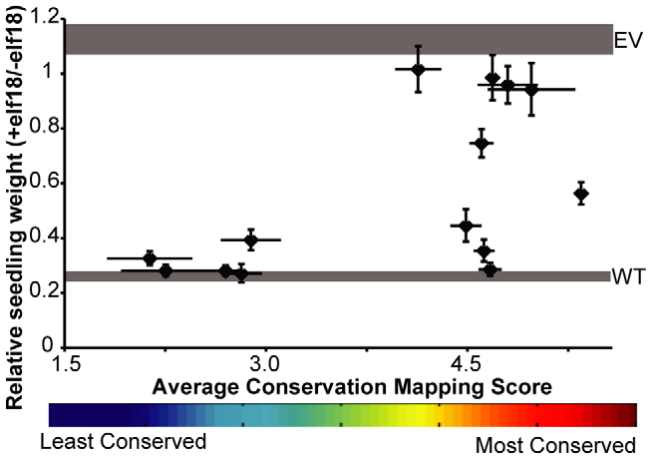
Functional testing of double-alanine mutagenesis alleles of EFR reveals that function-blocking mutations map to sites that RCM scores as conserved sites. A series of *EFR* alleles that each encode two alanine mutations within the β-strand/β-turn region of a single repeat (locations shown in Figure 4) were introduced into *efr^−^* plants and transgenic T1 seedlings were tested for their ability to respond to 100 nM elf18 in a seedling growth inhibition assay. Y-axis: Average weight of seedlings (+/− standard error), as compared to a no-peptide control for each line, for at least three replicate experiments with at least eight transgenic seedlings per genotype per treatment. Controls are weight (mean +/− standard error) of *efr^−^* seedlings transformed with wild-type *EFR* (WT, lower grey band) or empty vector (EV, upper grey band), as determined within the same experiments. X-axis: The average RCM regional conservation score for the two positions mutated in any given construct (x-axis bar ends for each symbol are at the RCM scores for the two positions mutated in each construct).

Hypothesizing that the ortholog map predicts functionally significant locations, we constructed a series of double-alanine mutants within EFR along the predicted concave face (also known as the β-strand, β-turn region [1]). For each construct, we changed two of the five variable residues within a single repeat's β-strand, β-turn region to alanine. Alleles were made that mutate sites of conservation, or as controls, sites lacking conservation. The double-alanine mutants were tested in stable transgenic *Arabidopsis efr^−^* plants. Of the fourteen constructs tested in a seedling growth inhibition assay, four of them had no detectable response to peptide, and four constructs had a response that was weaker than a wild-type response (Figure 5). Seven of these mutants were in conserved locations on the RCM map made from the EFR orthologs, including each of the four conserved patches of the β-strand, β-turn region (Figures 4, 5). The other two alleles with mutations in conserved locations did not detectably alter function. Importantly, only one of the five mutants in any of the poorly conserved regions resulted in a discernible difference in receptor function, with response slightly less than a wild-type receptor (Figures 4, 5). These results were further supported by ROS assays in *N. benthamiana* (not shown) and ROS and callose assays in *Arabidopsis* (Figure S4). All of the EFR double-alanine mutant proteins, whether functional or not, were still present at functional levels in plants, as detected by Western blot (Figure S5).

### Conservation mapping of EFR and its paralogs reveals a divergent β-strand, β-turn region

**Figure 6:**
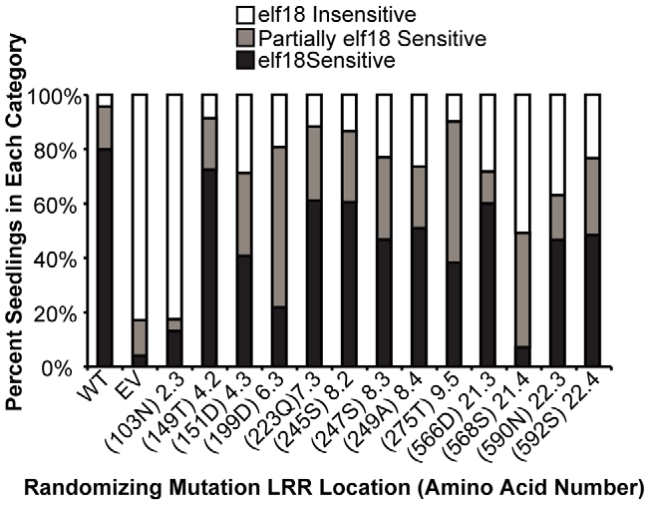
Site-directed, randomizing mutagenesis of EFR sites that are divergent in EFR paralogs reveals many partial-impact LRR sites. Seedling growth inhibition assays for EFR function were performed as in Figure 5 except that seedlings were treated with 1 uM elf18. A minimum of 65 T1 seedlings were tested for each allele library. Plants were classified as elf18-sensitive if their response was in the range of 95% of positive controls transformed with wild-type *EFR*, as elf18-insensitive if response was in the range of 95% of negative controls transformed with empty vector, or as partially sensitive if they fell within both ranges. WT: wild-type; EV: empty vector; allele number codes reflect the repeat number and the β-strand/β-turn position of the mutagenized amino acid (for example, 2.3 is the third x position of LxxLxLxxN in the second repeat), and parentheses enclose the amino acid number and letter.

Use of paralogs rather than orthologs was further examined. The four *Arabidopsis* paralogs most similar to EFR were identified by BLAST using the *Arabidopsis* EFR LRR sequence to query the *Arabidopsis* genome. An RCM map of EFR and these paralogs was then generated (Figure 4B). Some areas are generally conserved across these paralogs, in particular along one ‘shoulder’ of the convex face of the LRR that is also conserved among EFR orthologs. However, a large portion of the concave face of the LRR region is highly divergent in this paralog map. We hypothesized that the divergent β-strand, β-turn region is responsible for recognizing distinct ligands in these receptors, since 1) the concave face of LRR regions are most often implicated in ligand binding [15]; and 2) knock-out of *EFR* results in a plant completely insensitive to elf18 [32], implying that these EFR paralogs are not capable of EF-Tu recognition. Based on this map, we performed site-directed randomizing mutagenesis on *EFR* to create libraries of concave face mutations at amino acid positions that are highly divergent (predicted to impact function), or conserved (also predicted to impact function), or neither highly conserved nor highly divergent (not predicted to impact function). Each allele library contains different mutations at a single position. These libraries were introduced into *Arabidopsis efr^−^* plants that were then tested for response to 100 nM elf18 in a seedling growth inhibition assay. Most libraries were significantly impacted in their ability to recognize 100 nM elf18 (as compared to a wild-type receptor response) regardless of level of conservation, but all libraries still had several functional clones (Figure 6). The most significantly impacted library (LRR 2.3 (Asn 103)) was also identified in the double-alanine mutagenesis and is conserved in both RCM analyses performed (Figure 4). Overall, mutagenesis of regions conserved among EFR orthologs often broke function while mutagenesis of regions divergent among EFR paralogs had a less severe impact.

### Conservation mapping highlights an additional region of functional significance in AtFLS2

**Figure 7:**
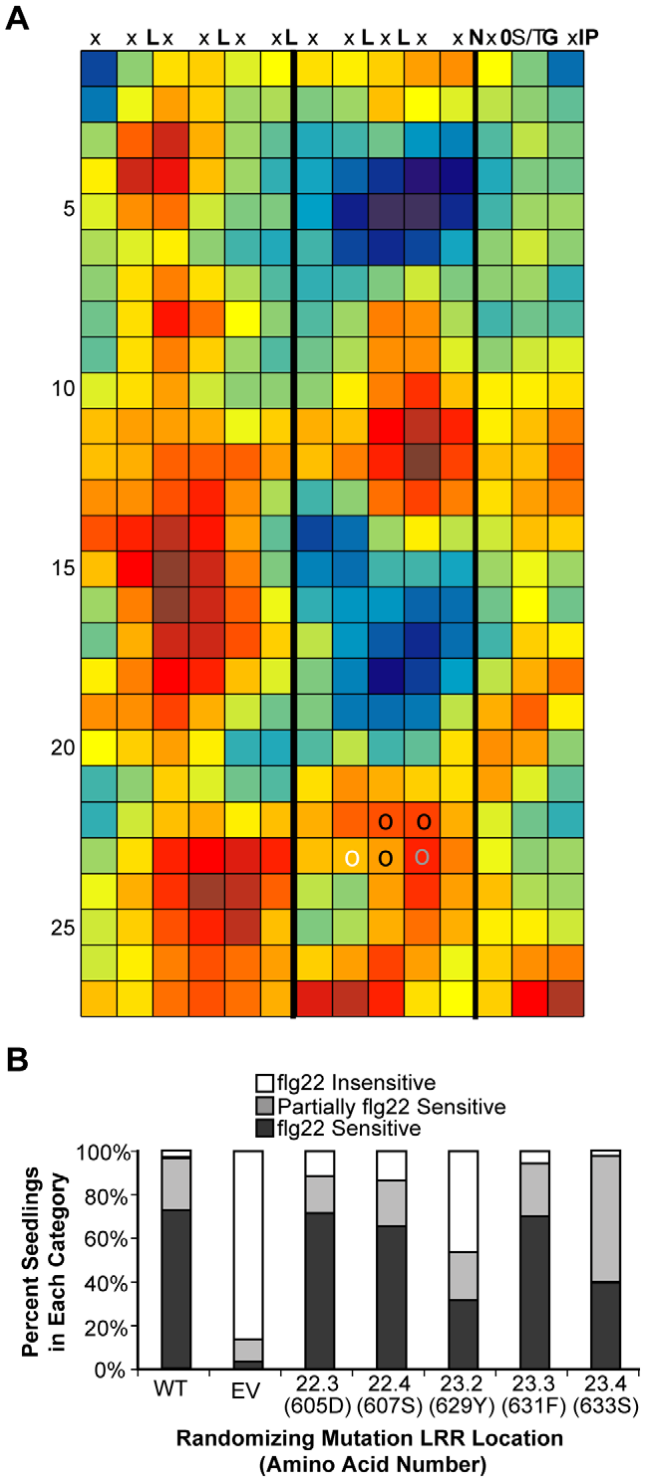
Site-directed, randomizing mutagenesis of five FLS2 residues in a region predicted by RCM to be functional identifies functional sites. (**A**) RCM map of eight Brassicaceae FLS2 orthologs. (**B**) Response to flg22 in T1 seedlings carrying FLS2 mutagenized at sites predicted by RCM. Each library represents a pool of *fls2-101* seedlings, each with one FLS2 construct carrying a random mutation at the position indicated. WT: wild-type; EV: empty vector; allele numbers as in Figure 6, and parentheses enclose the amino acid number and letter (indexed both by amino acid number and LRR position). Seedlings were scored as sensitive, insensitive, or partially sensitive to 1 uM flg22 in a seedling growth inhibition assay by comparison to 95% confidence intervals for data from *fls2-101* plants transformed with a wild-type *FLS2* or empty vector control, assayed on the same day. At least 65 plants per library were tested over 3–4 independent experiments, and the results are pooled.

RCM was further tested as a discovery tool through work with a second protein, FLS2. FLS2 has been deeply studied but there is no solved structure or definitively determined extracellular ligand binding site [33]. To identify additional important regions of the FLS2 LRR we created various RCM maps of AtFLS2, for example in comparison to eight Brassicaceae orthologs from plants that respond to flg22 (Figure 7A; see also [35]). Within the β-strand/β-turn region there were two main areas of conservation. One of these was located at LRRs 9–13, in agreement with previous findings that this is an important region for flg22 binding and recognition [35]. Another conserved area was persistently observed at LRRs 22–26. To investigate their role in flg22 perception, five solvent-exposed residues in this region were mutagenized by site-directed randomizing mutagenesis. These libraries were used to transform mutant *Arabidopsis fls2-101* plants that were then tested for response to flg22 peptide in a seedling growth inhibition assay. Alteration of Y629 significantly impacted flg22 responsiveness, and alteration of S633 also caused a detectable depletion of FLS2 activity (Figure 7B).

### RCM using many or few EFR and FLS2 orthologs, from closely or distantly related species

**Figure 8:**
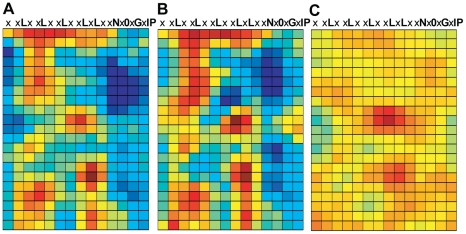
Conservation mapping using different EFR ortholog sets. (**A**) RCM map for ten Brassicaceae EFR orthologs. All sequences were confirmed as functional orthologs. (**B**) RCM map of two EFR orthologs, from distantly related *Eruca sativa* and *Erysimum raulinii*. (**C**) RCM map of two EFR orthologs, both from *Erysimum raulinii*. Maps are as described in Figure 2.

Changes in RCM output for a given protein, in response to varied types of input, was further examined after isolation of additional EFR orthologs from other Brassicaceae. EFR homologs were isolated by PCR from plant accessions that exhibited a response to elf18. A function in EF-Tu sensing was confirmed for nine homologous LRR domains (one each from *Brassica aucheri*, *Brassica rapa*, *Brassica napus*, *Eruca sativa*, *Biscutella auriculata* and two *EFR* sequences each from *Enarthrocarpus arcuatus* and *Erysimum raulinii*), using ROS assays for responsiveness to elf18 after transient expression in *N. benthamiana* leaves (data not shown). These confirmed homologs and the EFR sequence from *Arabidopsis thaliana* Col-0 were analyzed by RCM. The resulting map (Figure 8A) is notably similar to the map generated with only two EFR sequences (Figure 4). However, the map generated with ten sequences more clearly delineates the most consistently conserved clusters, which included the concave face regions confirmed to be required for EFR function (Figures 4, 5, 6).

Additional RCM maps were generated using smaller subsets of these EFR proteins based on the overall relatedness of different Brassicaceae species [83]. One analysis used EFR sequences from two of the most distantly related species of our sample set, *E. sativa* and *E. raulinii* (Figure 8B), while another analysis used two different EFR sequences obtained from a single *E. raulinii* plant (Figure 8C). A similar exercise was performed using FLS2 sequences from seven closely or distantly related Brassicaceae species (Figure S6). The maps of functionally similar LRRs from very closely related sequences (Figures 8 and S6) emphasize amino acid clusters containing less common amino acid residues that score highly in Blosum matrices, whether or not they reside at functional sites. The maps made from seven or ten functionally confirmed sequences more reliably emphasize evolutionary conservation of residue clusters. These latter maps also offer better resolution of conserved and diversified areas, but maps made from just two sequences from more distantly related species were sufficient to highlight the main features also present on the maps made from larger numbers of proteins (Figures 8 and S6, see also Figure 4). As with the map of *Arabidopsis* EFR and its paralogs, these ortholog maps also highlight regions outside of the concave face that have been conserved, presumably due to their functional significance.

### Comparison of conservation mapping to other computational methods

**Figure 9:**
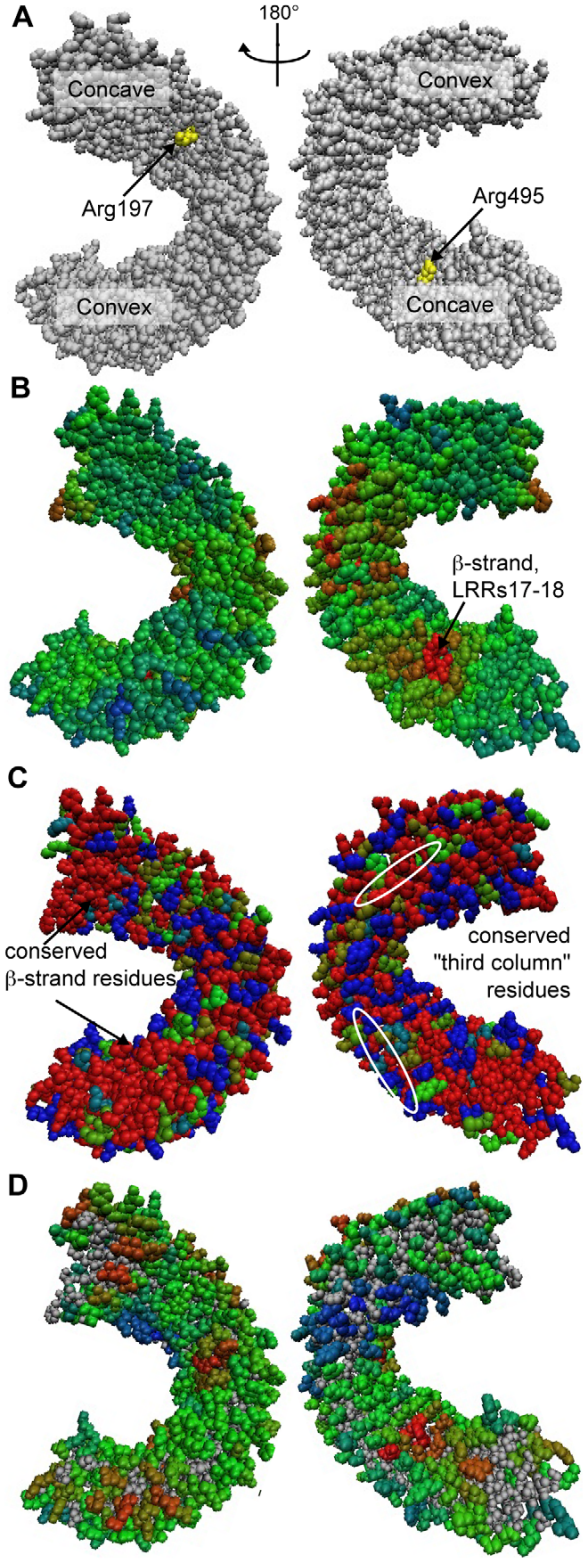
Analysis of EFR and orthologs by other computational methods. Results displayed on a homology model for (**A**) PAML positive/purifying selection analysis, (**B**) ODA analysis, (**C**) Consurf, and (**D**) RCM. For (A), residues undergoing positive selection (PP>.95 for at least one model) are highlighted in yellow. In (B), ODA exclusively utilized the homology model to make its functional predictions. For Consurf (C), derived amino acid sequence data for ten orthologous LRR domains from diverse Brassicaceae species were used (see text). To allow comparison, (D) presents the RCM data of Figure 4A on a homology model. Color scale ranges from “not predicted to be a binding site/not conserved” (blue) to “predicted to be a binding site/highly conserved” (red). All maps are shown in two views with 180° rotation between right and left columns; concave and convex faces of the LRR are indicated in (A). See Figure S7 for analysis of FLS2 using the same methods, and [55] for ODA and positive selection analyses of PGIP.

Other methods that predict important amino acid residues within a protein, beyond the BLAST or Pfam-like methods that identify conserved motifs in the primary sequence, include positive/purifying selection analysis [46], optimal docking area (ODA) calculation [55], and Consurf [49]. Because these three approaches employ different criteria for identifying functionally significant residues, we searched for important residues of EFR, FLS2 and PGIP using these three methods and compared their results to those generated by RCM (Figures 9 and S7). When a method required multiple sequences as input, we used the same sequences for each analysis (the Brassicaceae orthologs described above for EFR and FLS2, or the Fabaceae PGIP sequences from [55]). Homology models of EFR and FLS2 were created using the crystal structure of PGIP as template [56]. To allow comparison, the RCM results of Figure 4A are also presented on a homology model of EFR (Figure 9D).

As previously reported, integration of positive selection analysis and ODA identified several residues on the surface of PGIP that were important for specificity toward different PG's [55]. Unsurprisingly, most surface residues of EFR and FLS2, when compared to functional Brassicacea orthologs, were under purifying selection (−log(ω)>0). Only two residues of the LRR of EFR showed evidence of positive selection, neither of which is predicted to be on the concave face of the receptor (although both are on the surface of the protein) (Figure 9A). Similarly, two residues of the LRR of FLS2 are under positive selection, predicted to be on the side of the LRR and solvent-exposed (Figure S7).

Using ODA, a patch of potential ligand binding residues for EFR was identified on the β-strand, β-turn region of LRRs 17–18 (Figure 9B), overlapping with a region strongly identified by RCM. Two patches of predicted binding sites were detected for FLS2: a patch three to five surface residues wide in LRRs 1–5 occurring partially on the convex face and partially on the side of the LRR; and a patch primarily in the β-strand region of LRRs 10–15 (Figure S7). This second patch was previously shown to be important for flg22 recognition [35]. Consurf analysis of EFR highlighted many conserved areas; the most highly conserved residues were found in the second and third columns of the surface of the repeats as shown in Figure 4, and in the β-strand β-turn region (Figure 9C). Because the similarity of the Brassicacea FLS2 sequences is high (greater than 80%), Consurf ranked almost all residues as either highly conserved or highly divergent (Figure S7).

**Figure 10:**
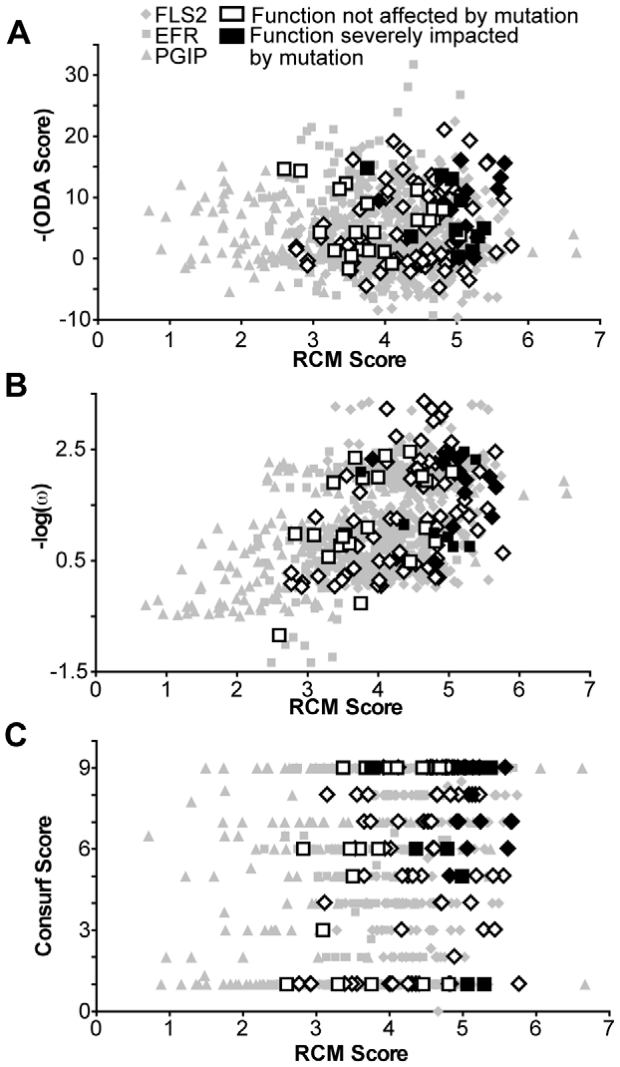
RCM analysis of FLS2, EFR, and PGIP performs as well as other computational methods. Y-axis: scores for each amino acid according to (**A**) positive selection analysis (nonsynonymous/synonymous substitution rates); (**B**) optimal docking area (scale is negative so that more likely docking sites are higher along y-axis); and (**C**) Consurf; all are graphed with respect to RCM score on the x-axis. Residues that were mutationally tested in the present study or in [35] are indicated as having no significant impact on function (white) or a significant impact on function (black) when mutated. Functional residues (in black) scored significantly higher than non-functional residues (in white) in all analyses completed (T-test; p<.05).

To investigate overlap between RCM output and outputs from the other computational methods, we looked for a correlation between our scoring method and positive selection analysis, ODA, and Consurf analysis (Figure 10). No strong correlation between any of the three computational methods and RCM was observed (R^2^<0.80). However, all methods could identify important functional residues. T-tests for differences between the set of scores for residues tested by mutation in which function was disrupted (Figure 10, black symbols), in comparison to the scores for residues for which function was retained after mutation (Figure 10, white symbols), revealed significant differences (p<0.05) for RCM, ODA, Consurf and positive selection analyses. However, of all the methods, RCM had the most significant difference (p<.0001). The ability of RCM to perform at least as well as other contemporary, well-accepted computational methods, in conjunction with its need for only two homologous sequences and no homology model, highlights the utility of this method.

## Discussion

The RCM method identifies regions on the predicted surface of LRR domains that have been conserved over the course of evolution. Conserved regions on the surface of folded proteins often correspond to key functional sites such as ligand binding sites or enzyme catalytic sites [44], [45]. In many cases where solved LRR+ligand crystal structures and appropriate homologs were available, RCM correctly identified the ligand binding regions of LRR domains. RCM also enabled discovery of previously unknown functional sites on the surface of EFR and FLS2. A strong trend was observed when RCM was used to direct mutational studies: significant impacts on function were frequently observed for mutations in regions that RCM predicted as conserved, while impacts were rare for mutations in non-conserved regions. This demonstrates the utility of RCM for *de novo* prediction of functional sites.

The RCM analyses of proteins for which there are LRR+ligand crystal structures demonstrated that conservation mapping can work for orthologs or paralogs, as long as they share similar functions. For example, creating a conservation map of RI orthologs highlighted functionally important regions previously demonstrated to be important for common function among these proteins. Mapping TIR1 and its *Arabidopsis* AFB1-5 paralogs, as well as PGIP1-4, demonstrated the utility of RCM for comparisons of functionally related paralogs. TIR1 functional sites again mapped to conserved regions of the protein. Irregularities in an LRR domain must be considered when generating and interpreting the results of RCM maps. Still, RCM performed well over irregular loop regions. In TIR1, for example, a loop region in the second LRR is involved in binding auxin, and RCM correctly identified this region as a functional site.

In contrast to comparison of orthologs, functionally important sites of PGIP could be seen in divergent regions highlighted by the program, as might be expected since PGIPs display varied specificity and inhibition mechanisms towards different polygalacturonases [84], [85]. This reinforces the point that careful choice of input sequences is crucial to gaining meaningful output data. It will be interesting in the future to map plant NB-LRR proteins that exhibit direct recognition of changing pathogen ligands, where the most divergent LRR sites might be predicted to be the sites of ligand recognition, and to compare these results to those obtained using Ka/Ks (positive selection) analyses (e.g., [86], [87]).

Although it was encouraging that 45% of all interaction residues from LRR+ligand structures appeared in the top 20% of RCM scores, some interaction residues received low conservation scores in RCM, indicating that these positions are not conserved. These amino acids could be responsible for differences in specificity of the compared proteins rather than any shared activity. Even in cases where two related proteins have extremely similar functions, some variable residues are to be expected at functional sites as these could be responsible for fine-tuning of receptor function, such as through ligand interaction kinetics. As a separate issue, there were many residues that scored highly in RCM that did not interact with ligand in the available crystal structure. While these could be false positives, many of them are likely responsible for functions not detectable in the crystal structure (i.e., interaction with molecules not present in the crystal structure). The regions highlighted by RCM but not matching known LRR+ligand interaction sites are intriguing targets for future study.

In LRR domains, disruption of single solvent-exposed residues very frequently does not disrupt function (e.g., [35], [88]). We were pleased to find seven different double-alanine mutants of EFR that were each severely disrupted in function when compared to a wild-type receptor. These mutants were in four distinct conserved regions on the surface of EFR, one of which overlaps with a region identified by [36] as important for EFR function. A full-length protein can be detected for these different EFR LRR mutation alleles (Figure S5), but that leaves many other possible activities that could be disrupted. Some of these patches may fold together to form a shared ligand-binding domain, but alternatively, three of the four sites may be involved in other processes such as receptor dimerization, interaction with co-receptors, or protein localization. Experiments are in progress to investigate the functions that are disrupted when these different regions are mutated. One prediction is that different function-blocking mutations in the same region (such as in the concave face of LRRs 11–12, or LRRs 16–17) will cause the same type of functional disruption.

Interestingly, although seven of the nine double-alanine mutants made in conserved EFR regions disrupted function, two of the mutants behaved in a manner identical to wild-type. This demonstrates that not all residues in a conserved region are essential for function. It is important to note that the score placed at any residue position is a regional conservation score for a 5×5 window, and may be elevated due to the presence of multiple conserved residues surrounding a relatively less conserved center residue. Examination of the single-residue conservation scores (as in Figure S2) may help to identify the most significant residues in a conserved region. Alternatively, the conserved regions not functionally disrupted by mutations may be functionally important in ways that would be not detected using the ligand that we utilized. As a third option, it is also possible that RCM detects a certain amount of ‘noise’ that represents relatively unselected stochastic conservation.

Prediction of functionally significant EFR sites was originally performed with only two sequences. When we identified a further seven functional orthologs of EFR and created a conservation map with these additional sequences, the same sites were identified, probably with more precision. Use of two sequences could be misleading when the two sequences are very similar (see also the FLS2 maps of Figure S6). Additional sequences may be a means of increasing the power or reliability of RCM, but even a pairwise comparison can be informative.

We chose to focus on the β-strand, β-turn region of EFR because of the propensity of ligand binding sites to occur in this region [15]. However, in a few cases crystal structures have identified important residues outside the concave face of the protein, such as in the case of TLR3. The regions outside the concave face that were highlighted by the present conservation mapping effort may also be important for receptor function. For example, [89] recently identified a site of glycosylation, N143, on the convex face of the LRR of EFR that, when mutated, results in a severely impaired receptor. This residue occurs in the fourth repeat within the large conserved patch identified in both the ortholog and paralog conservation maps of EFR (Figure 4).

We were surprised that the most divergent region RCM identified among related but functionally distinct EFR paralogs, centered on the concave face of the fifth through ninth repeats, contained residues with an intermediate rather than a strong impact elf18 perception (Figure 4B and Figure 6). We infer that these paralogs are functionally distinct from EFR because none of the four paralogs confer elf18 recognition when they remain wild-type in an *efr^−^* mutant. This failure to see a strong impact on EFR function, when testing many of the site-directed random mutations of single residues in this most divergent region, may have occurred because the divergent sites we tested are more important for the gain of function of one or more of the other paralogs while being relatively unimportant for the responsiveness of EFR to EF-Tu. It is equally possible that these divergent regions carry many residues that each make only modest contributions to elf18 recognition, with tolerance for alternative amino acid side chains. Alternatively, as was discussed for the double-alanine mutations, insignificant residues within functional regions may have been chosen for testing in our non-exhaustive mutational screen, or it may be unselected chance that the LRRs of EFR and its paralogs have diverged in this area. If these regions are under no substantial selective pressure one might expect that they would show intermediate coloration in the RCM image rather than deep blue. The deep blue sites on the map of Figure 4B are in fact quite diverse; the set of five EFR paralogs carried at least three different amino acids at most of the mutated positions, and in many cases five. As a separate matter, in the future it will be interesting to test the large conserved region that occurs on the convex face of the LRR region, especially since this region is conserved among both orthologs and paralogs of EFR.

Repeat conservation mapping offers advantages over other methods that could be used to identify functional regions. RCM only requires two homologous sequences to compare, whereas positive selection analysis and Consurf are best done with at least seven sequences [46], [49]. Most methods that utilize information about the spatial proximity of residues in a folded protein, such as ODA, CFG and Consurf, require a solved crystal structure or a homology model. However, LRR domains can be very challenging to crystallize, and generation of a valid homology model is also highly challenging. The validation experiments conducted with RCM suggest that use of a generic LRR structural model with removal of consensus residues is sufficient to successfully predict areas of conservation or divergence.

There were significant instances of overlap in the functional regions predicted by ODA and RCM. However, the two methods rely on very different concepts and they also identified non-overlapping regions in response to the same input data. This included functionally confirmed regions such as the LRR11 region of EFR and the LRR 23 region of FLS2 that ODA did not identify. Consurf was not highly informative when used with our homology-modeled FLS2 or EFR (Figures 9 and S7), possibly because of the proximity of many LRR consensus sequence residues near the LRR surface. The CFG program, while previously shown by us to be useful for some parts of LRRs [35], is not optimal because it removes hydrophobic residues from consideration even if they are on the protein surface. CFG also takes into account residues that are in the consensus of many LRRs, such as the N and P residues that are largely buried and highly conserved for structural reasons rather than driving functional differences between LRRs. By assessing variable-position rather than consensus-position residues of the LRR, RCM may benefit from a focus on the residues that are most likely to impact function for reasons other than overall protein structure and integrity.

Future improvements to RCM are anticipated. Output of results onto a generic 3D LRR model as a pdb file may be useful, for viewing and manipulation in PyMOL, Swiss-PdbViewer or similar programs. The LRR repeat models developed in an RCM run currently require hand-curation, but greater automation of LRR matrix building is anticipated, for instance through use of HMMER (http://hmmer.janelia.org) to build consensus logos of the proteins being compared. RCM at this stage is limited to use with LRR domains, but because RCM utilizes typical repeat domain structures rather than precise spatial data, the method is likely to be adaptable to other repeat proteins that have a known repetitive structure. This includes armadillo, tetratricopeptide, and ankyrin repeats. Adaptation of conservation mapping to ankyrin repeats could be particularly insightful, as these domains are the focus of artificial evolution towards novel ligands [90].

The repeat conservation mapping approach is a predictive method that can be utilized to identify key similarities and differences among groups of homologous LRR-containing proteins. The RCM program predicts functional sites in LRR domains, which may facilitate basic structure-function studies or *in vitro* protein evolution toward modified functions for these widespread and biologically significant domains. The current implementation is functional for LRRs, but it should be modifiable for use with other repeat-containing protein domains.

## Supporting Information

Figure S1 **Additional RCM maps for LRR domains for which there are solved LRR+ligand structures.**
(PDF)

Figure S2 **Maps of individual residue conservation scores, at an intermediate step in RCM prior to calculation of regional conservation scores.**
(PDF)

Figure S3 **Identification of an EFR ortholog.**
(PDF)

Figure S4 **Examples of ROS and callose assays of** ***EFR*** **double-alanine mutant alleles expressed under control of native EFR promoter in transgenic** ***Arabidopsis*** **T1 seedlings.**
(PDF)

Figure S5 **Presence of EFR-HA double-alanine mutant proteins, whether functional or not, in transgenic** ***Arabidopsis*** **seedlings.**
(PDF)

Figure S6 **RCM maps of FLS2 generated using different FLS2 ortholog sets.**
(PDF)

Figure S7 **Analysis of FLS2 and PGIP by other computational methods.**
(PDF)

Text S1 **Description of the RCM method.**
(DOC)
