# Supplementary material for: LRR Conservation Mapping to Predict Functional Sites within Protein Leucine-Rich Repeat Domains
Source: PLoS One. 2011 Jul 18;6(7):e21614. doi: 10.1371/journal.pone.0021614 (PMC3138743; doi:10.1371/journal.pone.0021614)

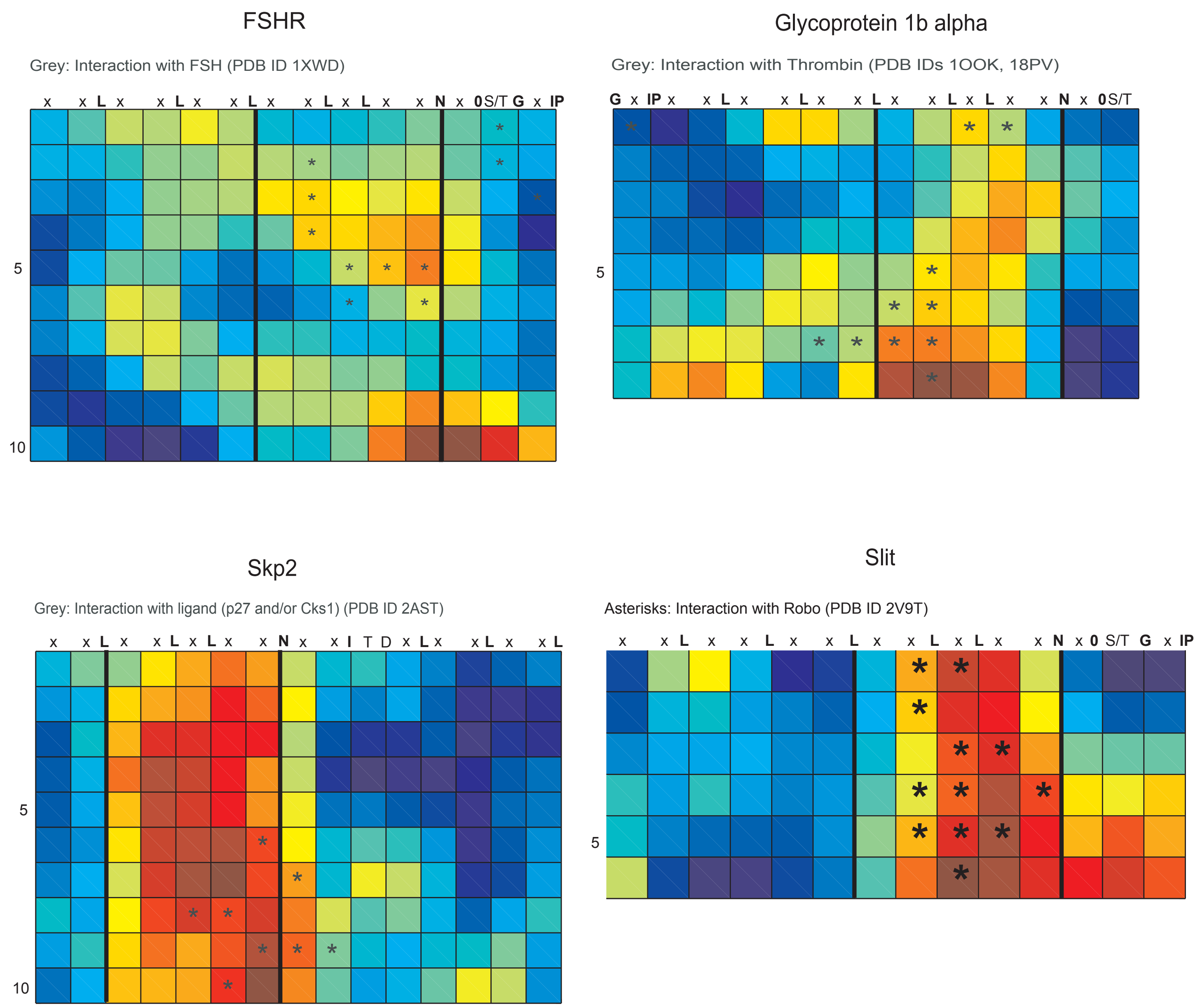

Figure S1: Additional RCM maps for LRR domains for which there are solved LRR+ligand structures. For all maps, each row represents a single repeat of the LRR, with each colored box representing a solvent exposed (non-consensus) amino acid position. Each column corresponds to a position within the LRR consensus sequence, as denoted at the top of each map. The color in each box reports the center-weighted regional conservation score for the 5x5 set of boxes that centers on that box (see text for details); red indicates the most conserved regions and blue indicates the most divergent regions (see scale bar in Fig. 1). Bold black vertical lines delineate the five residues in each row that comprise the β-strand, β-turn region (the convex face of the LRR domain). The asterisks were added after RCM and indicate amino acid positions that are LRR-ligand contact points in solved crystal structures.

TLR1

Grey: Interaction with ligand (PDB ID 2Z7X)
Black: Interaction with TLR2 co-receptor (PDB ID 2Z7X)

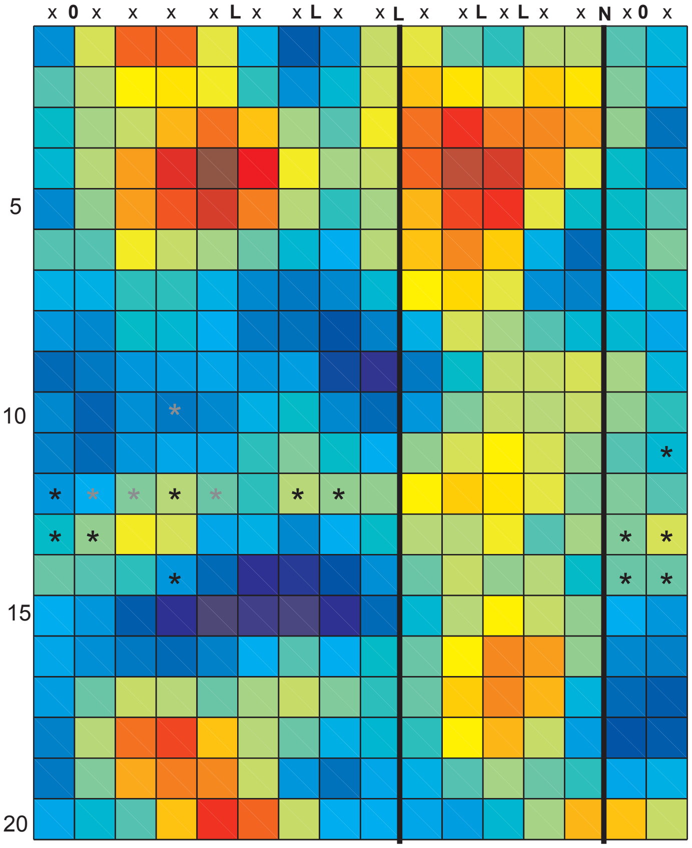

TLR2

Black: dimerization with TLR1 or TLR6 (some also interact with ligand) (PDB IDs 2Z7X, 3A7B, 3A7C, 3A79)

Grey: interaction with lipid ligand (specificity determining) (PDB IDs 2Z7X, 3A7B, 3A7C, 3A79)

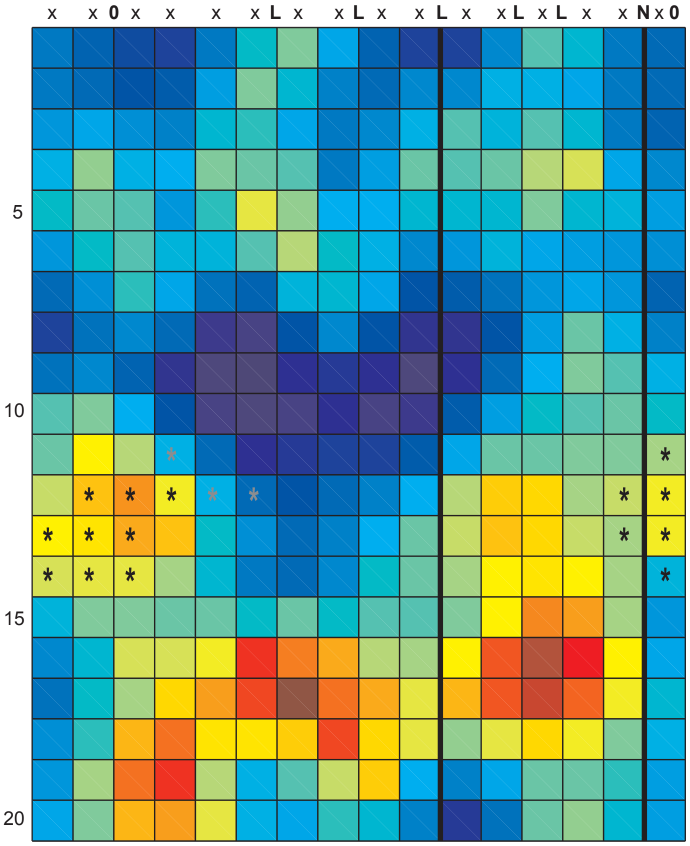

TLR4

Black: Interaction with MD-2 (conserved function) (PDB IDs 3FXI, 2Z64)
Grey: Interaction with LPS (specificity) (PDB ID 3FXI)

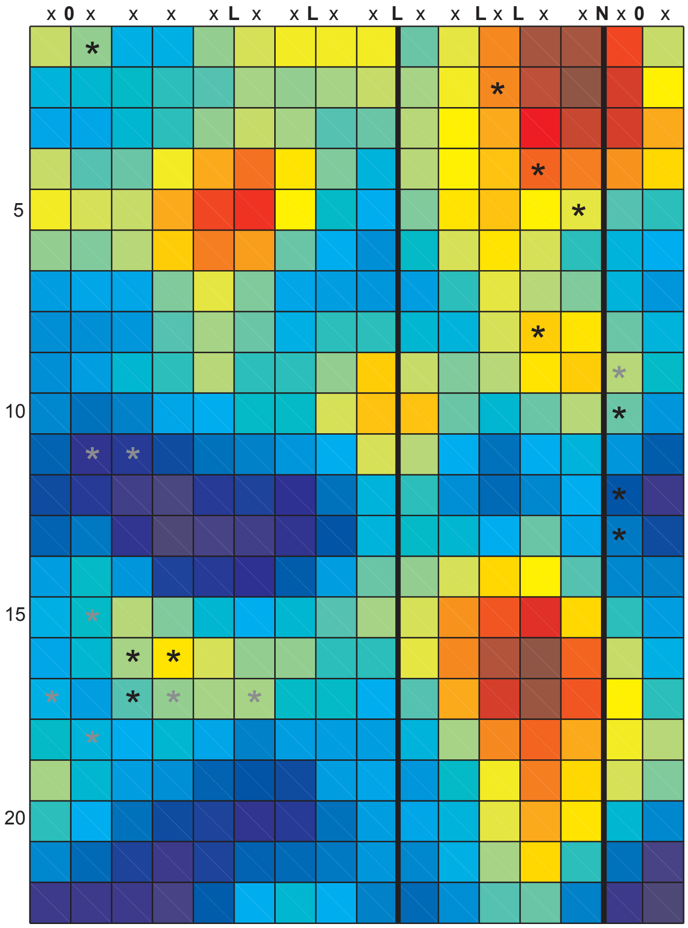

TLR3

Grey: Interaction with dsRNA (PDB ID 3CIY)

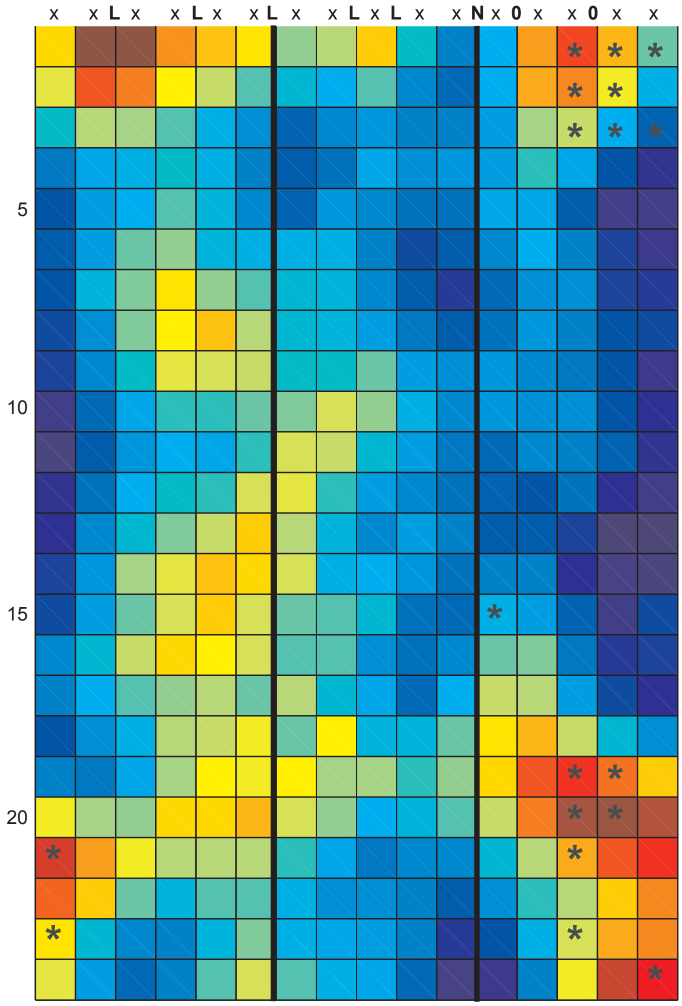

TLR6

Grey: Interaction with ligand (PDB ID 3A79)
Black: Interaction with TLR2 co-receptor (PDB ID 3A79)

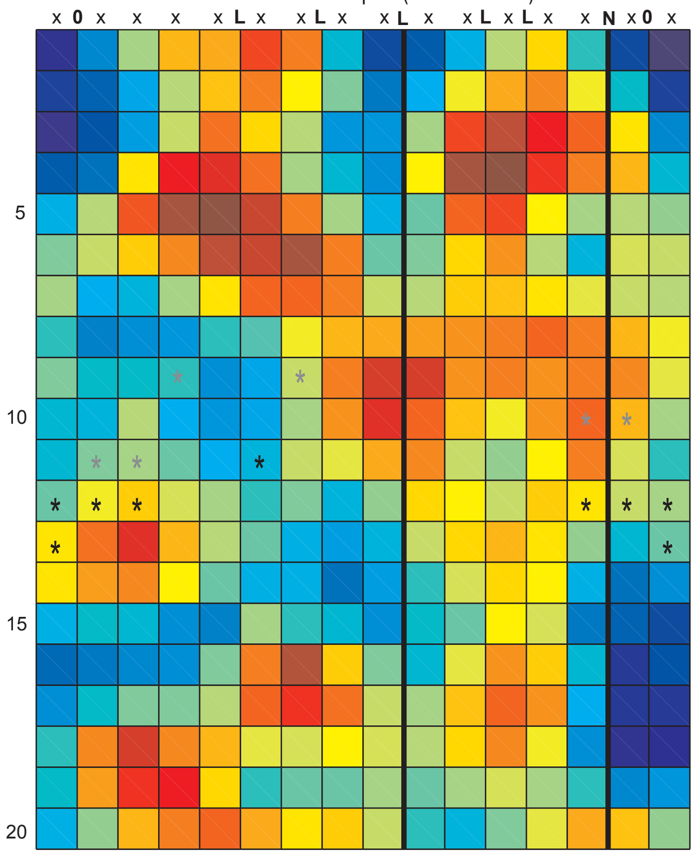

Supplement: Figure S1 — Additional RCM maps for LRR domains for which there are solved LRR+ligand structures. (PDF) [file pone.0021614.s001.pdf]
