# Supplementary material for: LRR Conservation Mapping to Predict Functional Sites within Protein Leucine-Rich Repeat Domains
Source: PLoS One. 2011 Jul 18;6(7):e21614. doi: 10.1371/journal.pone.0021614 (PMC3138743; doi:10.1371/journal.pone.0021614)
Supplement: Figure S2 — Maps of individual residue conservation scores, at an intermediate step in RCM prior to calculation of regional conservation scores. (PDF) [file pone.0021614.s002.pdf]

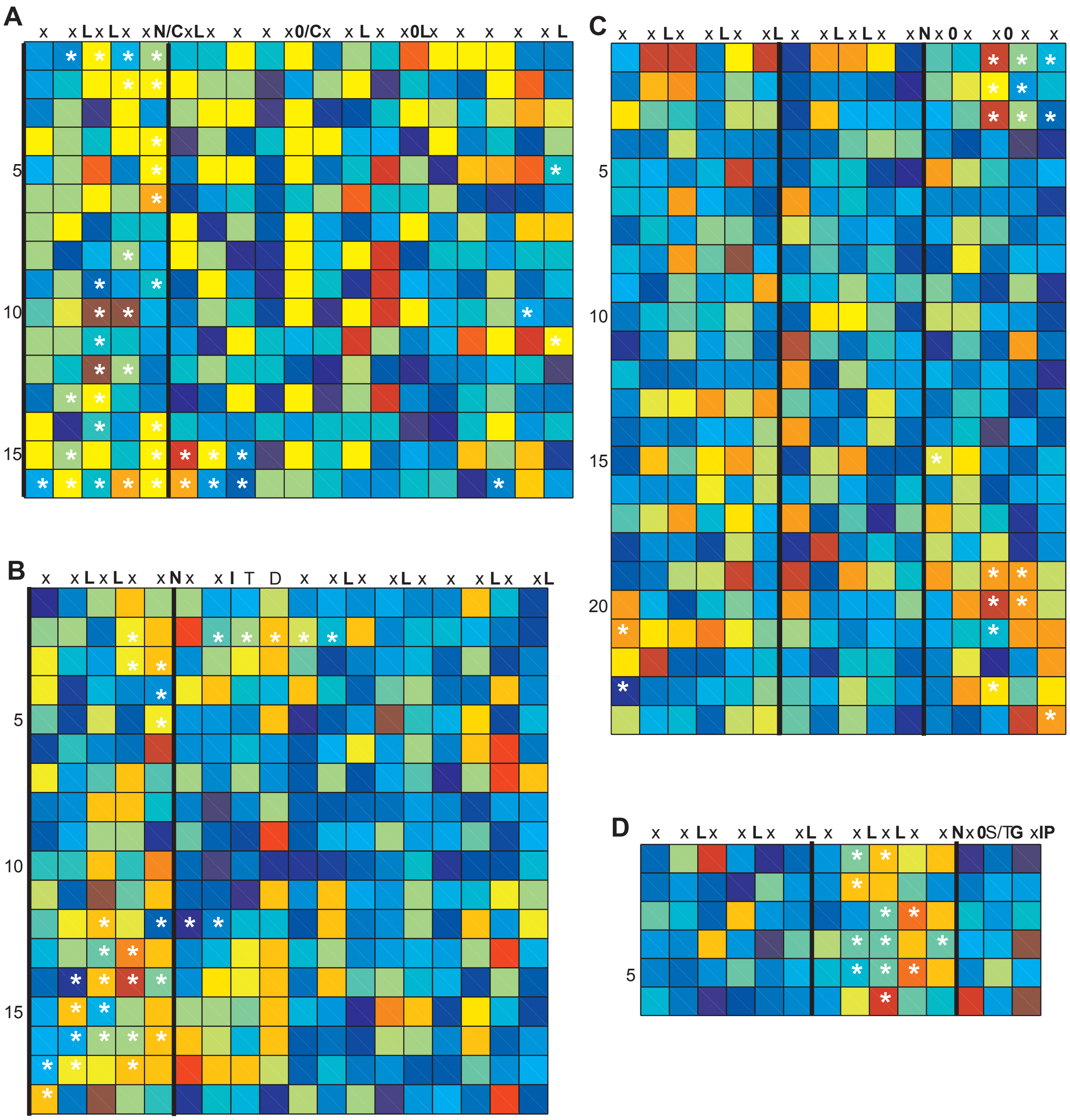

Figure S2: Maps of individual residue conservation scores, at an intermediate step in RCM prior to calculation of regional conservation scores. The residue conservation score diagrams in this figure (which are not full RCM maps) were made using the input data used for the RCM maps in Figures 2 and S1, for A) ribonuclease inhibitor, B) auxin receptor (TIR1 and AFB1-5), C) TLR3,and D) slit.
