# Supplementary material for: LRR Conservation Mapping to Predict Functional Sites within Protein Leucine-Rich Repeat Domains
Source: PLoS One. 2011 Jul 18;6(7):e21614. doi: 10.1371/journal.pone.0021614 (PMC3138743; doi:10.1371/journal.pone.0021614)
Supplement: Figure S3 — Identification of an EFR ortholog. (PDF) [file pone.0021614.s003.pdf]

A

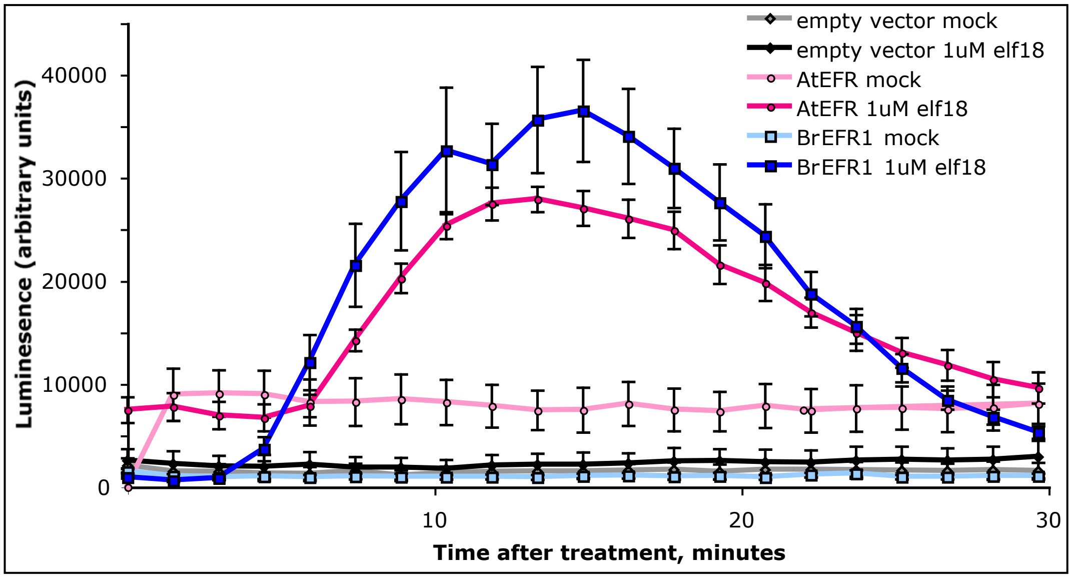

B

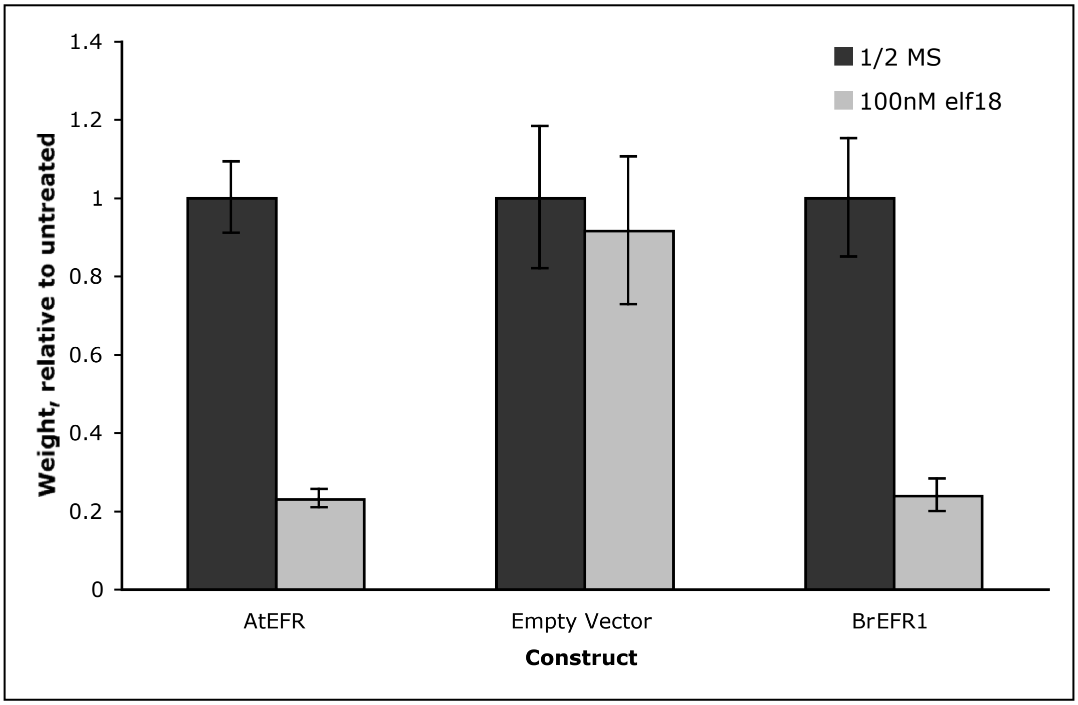

Figure S3: Identification of an EFR ortholog. Two high-scoring BLAST hits (highly similar to Arabidopsis EFR) were identified at the Brassica Genome Gateway, both from *B. rapa.* One of these was cloned by PCR (see Methods), fully sequenced, and expressed in *N. benthamiana* (see Methods). This sequence, designated BrEFR1, conferred elf18 sensitivity in an ROS assay when A) transiently expressed in *N. benthamiana* leaves, and B) rescued elf18 perception as seen in a seedling growth inhibition assay when expressed in stable transgenic T1 seedlings derived from an Arabidopsis efr mutant line.
