# Supplementary material for: LRR Conservation Mapping to Predict Functional Sites within Protein Leucine-Rich Repeat Domains
Source: PLoS One. 2011 Jul 18;6(7):e21614. doi: 10.1371/journal.pone.0021614 (PMC3138743; doi:10.1371/journal.pone.0021614)
Supplement: Figure S4 — Examples of ROS and callose assays of EFR double-alanine mutant alleles expressed under control of native EFR promoter in transgenic Arabidopsis T1 seedlings. (PDF) [file pone.0021614.s004.pdf]

A

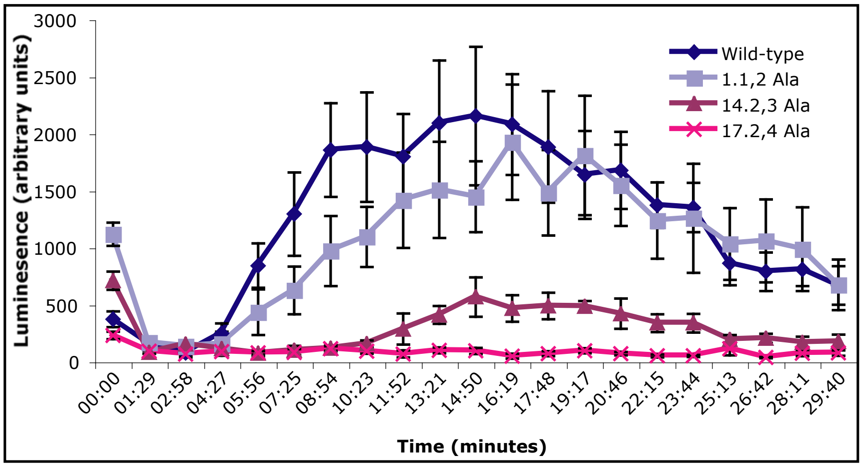

B

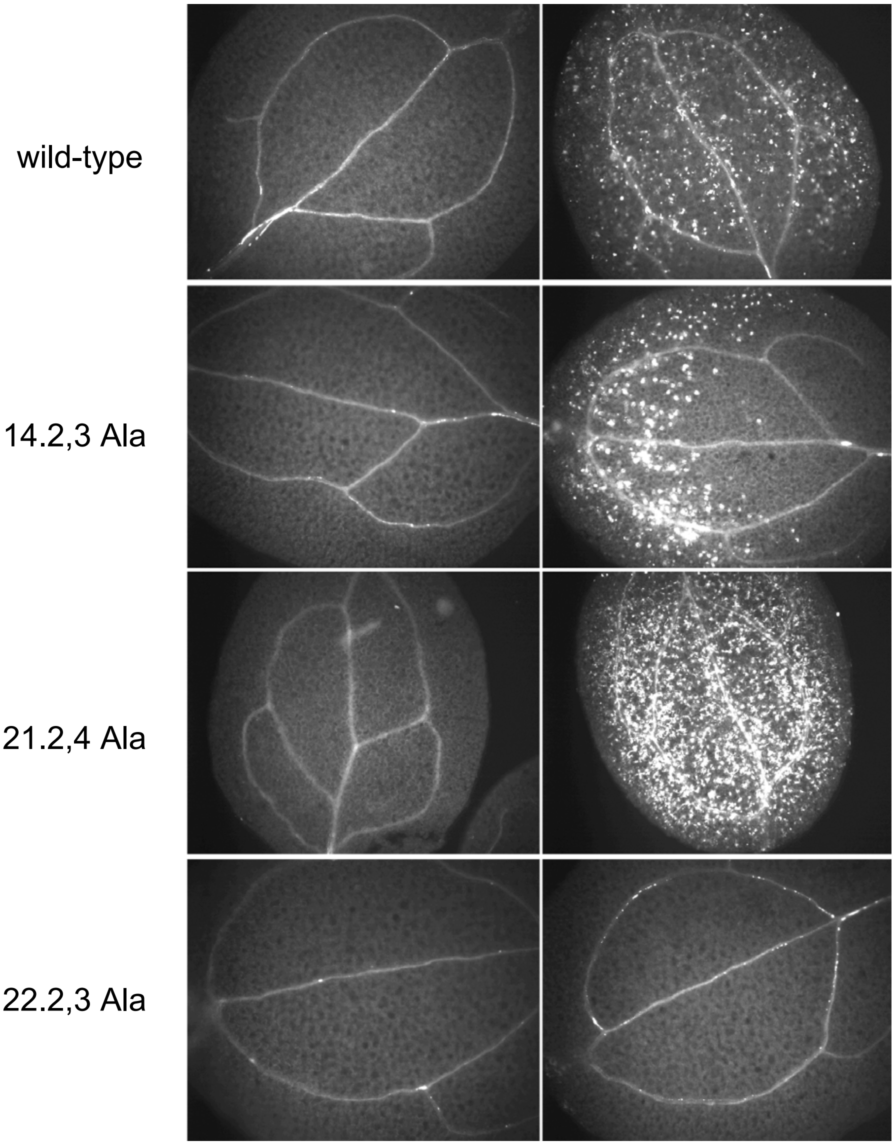

Figure S4: Examples of ROS and callose assays of EFR double-alanine mutant alleles expressed under control of native EFR promoter in transgenic *Arabidopsis* T1 seedlings.
