# Supplementary material for: LRR Conservation Mapping to Predict Functional Sites within Protein Leucine-Rich Repeat Domains
Source: PLoS One. 2011 Jul 18;6(7):e21614. doi: 10.1371/journal.pone.0021614 (PMC3138743; doi:10.1371/journal.pone.0021614)
Supplement: Figure S5 — Presence of EFR-HA double-alanine mutant proteins, whether functional or not, in transgenic Arabidopsis seedlings. (PDF) [file pone.0021614.s005.pdf]

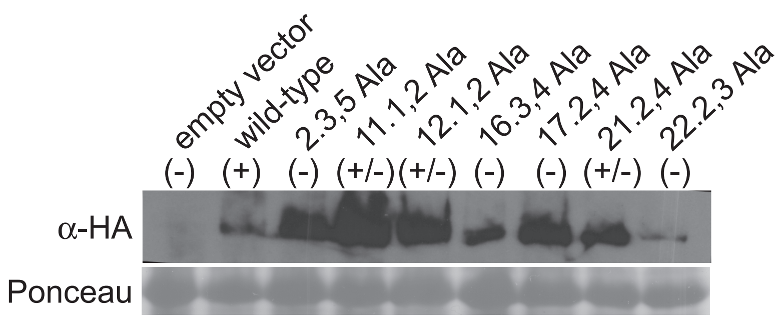


| construct | mutated amino acids | seedling growth inhibition | reactive oxygen species | callose |
|---|---|---|---|---|
| wild-type | N/A | ++ | ++ | ++ |
| empty vector | N/A | - | - | - |
| 1.1,2 Ala | I76, S77 | ++ | ++ | ++ |
| 1.4,5 Ala | G81, G82 | ++ | ++ | ++ |
| 2.3,5 Ala | N103, D106 | - | - | - |
| 9.4,5 Ala | G274, T275 | ++ | ++ | ++ |
| 10.1,2 Ala | E293, R294 | ++ | ++ | + |
| 10.4,5 Ala | S298, S299 | ++ | ++ | ++ |
| 11.1,2 Ala | W317, W318 | + | + | + |
| 12.1,2 Ala | E347, W348 | + | + | ++ |
| 13.2,3 Ala | S373, F375 | ++ | ++ | ++ |
| 14.2,3 Ala | E397, S399 | + | + | + |
| 16.3,4 Ala | H447, N449 | - | - | - |
| 17.2,4 Ala | D469, D473 | - | - | - |
| 21.2,4 Ala | N564, S568 | + | + | ++ |
| 22.2,3 Ala | N588, N590 | - | - | - |

**Figure S5: Presence of EFR-HA double-alanine mutant proteins, whether functional or not, in transgenic *Arabidopsis* seedlings.** Gel lanes were equally loaded for total protein, as confirmed by Ponceau stain, and detected using anti-HA on Western blots after SDS-PAGE. A table is also presented, summarizing results with these mutant alleles.
