# Supplementary material for: LRR Conservation Mapping to Predict Functional Sites within Protein Leucine-Rich Repeat Domains
Source: PLoS One. 2011 Jul 18;6(7):e21614. doi: 10.1371/journal.pone.0021614 (PMC3138743; doi:10.1371/journal.pone.0021614)
Supplement: Figure S6 — RCM maps of FLS2 generated using different FLS2 ortholog sets. (PDF) [file pone.0021614.s006.pdf]

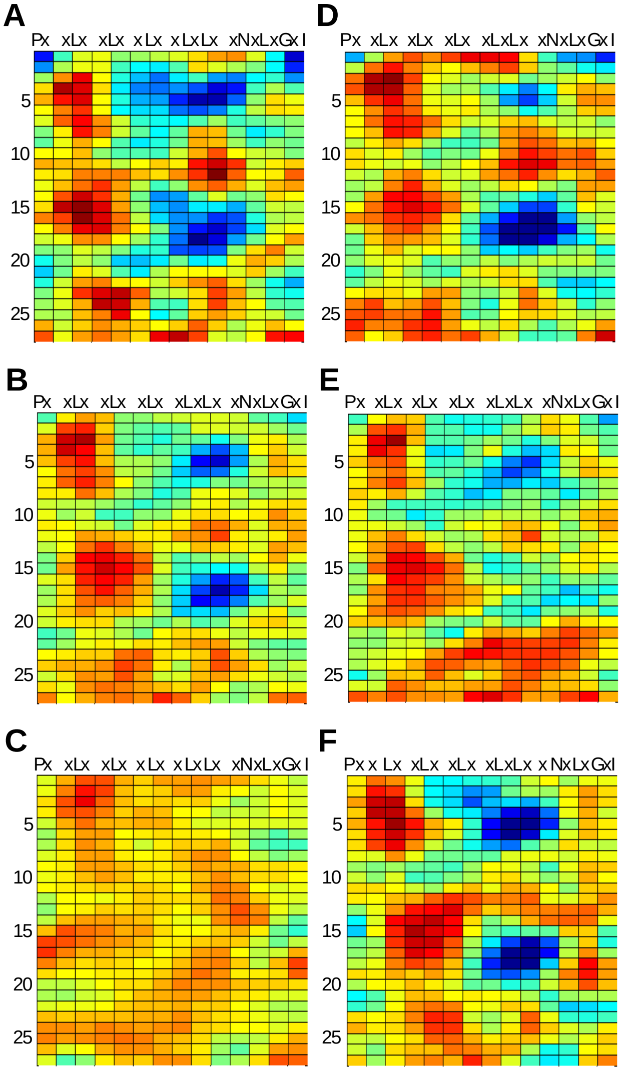

Figure S6: RCM maps of FLS2 generated using different FLS2 ortholog sets. Input was derived amino acid sequences from: A) Seven Brassicaceae species, similar to print Figure 8. B) One representative each from Brassicaceae lineages I, II and III as in Couvreur et al. 2010 (*Camelina laxa, Eruca vesicaria* and *Chorispora tenella*). C) Two highly similar FLS2 isolates, both from *Brassica rapa*. D), E) and F) Two FLS2 isolates are compared, from: D) lineages I and II *Camelina laxa* and *Eruca vesicaria*; E) lineages I and III *Camelina laxa* and *Chorispora tenella*; F) lineages II and III *Eruca vesicaria* and *Chorispora tenella*. Maps are described in print Figure 2.
