# Supplementary material for: LRR Conservation Mapping to Predict Functional Sites within Protein Leucine-Rich Repeat Domains
Source: PLoS One. 2011 Jul 18;6(7):e21614. doi: 10.1371/journal.pone.0021614 (PMC3138743; doi:10.1371/journal.pone.0021614)
Supplement: Figure S7 — Analysis of FLS2 and PGIP by other computational methods. (PDF) [file pone.0021614.s007.pdf]

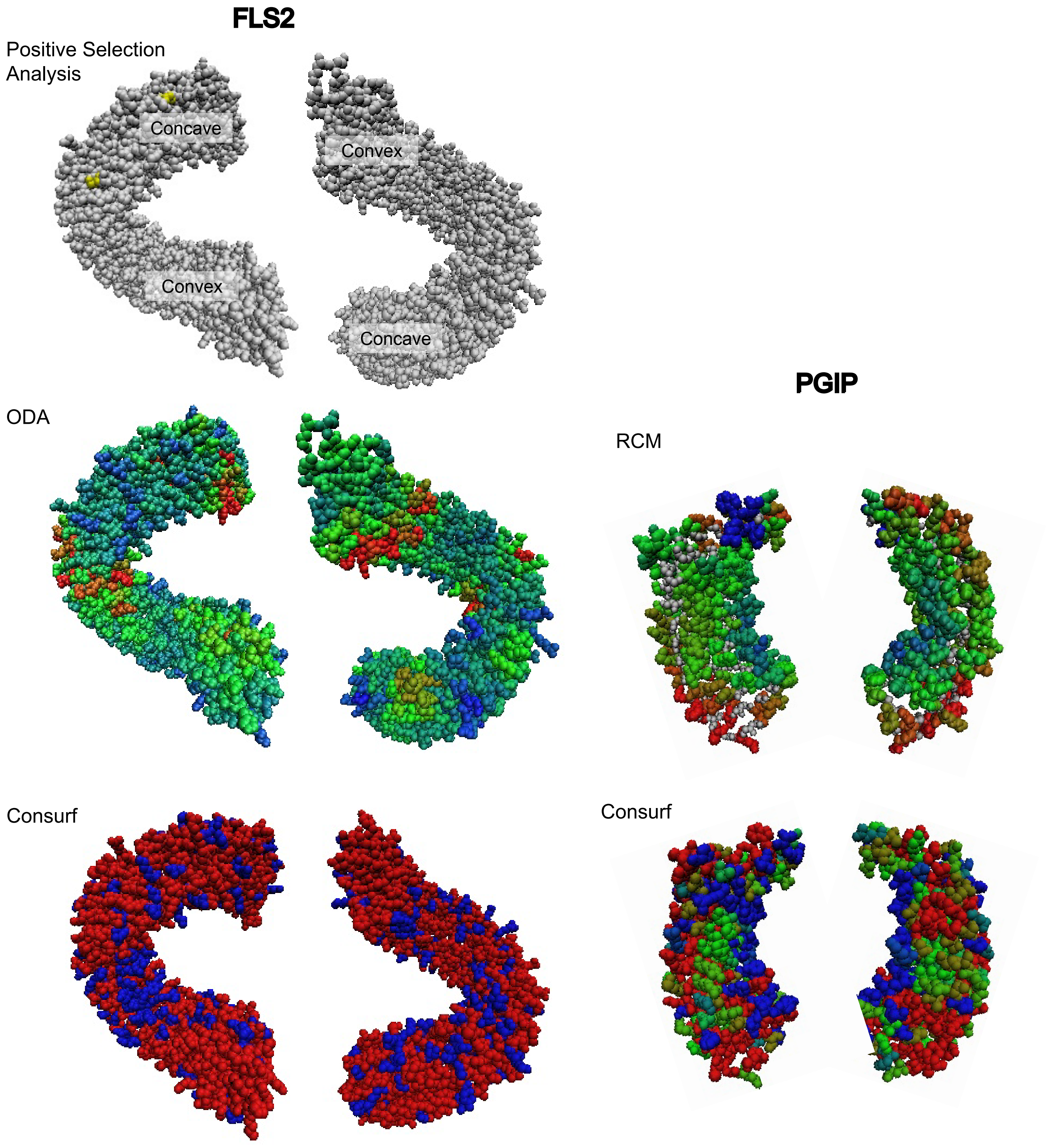

Figure S7: Analysis of FLS2 and PGIP by other computational methods. Positively selected (divergent) sites on the predicted FLS2 LRR are shown in yellow. Color scales for ODA and Consurf results are the same as in Figure 9. A homology model for the FLS2 LRR domain was used as input for ODA and Consurf analyses, along with derived amino acid sequence data for 10 FLS2 LRR domains from diverse flg22-responsive Brassicaceae species (see text). Output from positive selection (performed on the linear amino acid sequences) was mapped onto the homology model for comparison. All maps are shown in two views with 180[illegible] rotation between right and left columns.
