# Supplementary material for: LRR Conservation Mapping to Predict Functional Sites within Protein Leucine-Rich Repeat Domains
Source: PLoS One. 2011 Jul 18;6(7):e21614. doi: 10.1371/journal.pone.0021614 (PMC3138743; doi:10.1371/journal.pone.0021614)
Supplement: Text S1 — Description of the RCM method. (DOC) [file pone.0021614.s008.doc]

**Supplemental Text File S1: Description of the RCM method**

***Step 1: Identify homologous proteins***

One or more homologs (either orthologs or paralogs) of a protein of interest are required as input for RCM analysis. BLAST algorithms or similar methods can be deployed to search databases, preferably using the LRR domain alone as query, to identify sequences similar to the query sequence. Investigators may choose to isolate their own sequences for comparison to the query protein. When available, sequences of orthologous proteins known from experimental evidence to have similar function to the protein of interest are an excellent input; using such sequences allows informed conclusions about conserved regions.

***Step 2: Identify LRR domains***

Initial discovery of LRR-containing regions can be accomplished by use of the conserved domain identification algorithms associated with online NCBI BLAST implementations, or using Pfam or other widely available methods. LRR domains may be more completely identified by manual user editing or by use of LRRScan [53]. In any case, quality control by manual editing is desirable as current algorithms often successfully identify some but not all repeat units of LRRs within a protein, or over-identify LRRs if search criteria are not sufficiently stringent.

***Step 3: Align proteins***

LRR domains and their immediately flanking regions are aligned within RCM, using ClustalW2 algorithms with the default settings. Output scores for overall similarity are saved for calculation of conservation in Step 4.

***Step 4: Score conservation of each position in alignment***

Conservation at each amino acid position is calculated as a weighted average of BLOSUM65 scores for pairwise comparisons of all sequences in the alignment, with weighting inversely proportional to the ClustalW2-determined overall similarity between the two sequences being compared (done to dilute the effects of very similar proteins and enhance the effect of more distantly related proteins). Several different BLOSUM and PAM matrices have been tested for scoring and tend to give similar overall RCM results, except that the distribution of scores (and therefore the contrast of the maps) varies depending on the matrix used.

***Step 5: Arrange LRRs into two-dimensional matrix***

Individual repeats, identified in Step 2, are used together with LRRScan and ClustalW2 algorithms to build a matrix of amino acid positions that represent the entire LRR. The predicted LRR arrangement is displayed for users and an opportunity for manual editing is offered. Each row of the matrix is one full repeat (typically 22-26 amino acids) within the LRR. Due to the tertiary conformation naturally adopted by LRRs, alignment of the multiple repeats causes each column of the matrix to carry amino acid positions that are predicted to align vertically across repeats in the actual folded LRR protein. Each cell within the matrix is indexed both to the residue identities for that position and to the conservation score for the aligned residues at that position (as calculated in Step 4).

***Step 6: Eliminate consensus residues from analysis***

In essentially all known crystal structures of LRRs the LRR consensus residues are primarily buried rather than solvent exposed. These consensus residues are crucial in specifying the overall solenoid shape of an LRR domain but they are quite similar across diverse LRR proteins, while the determinants of functional specificity within families of related LRR domains reside primarily on the surface. Furthermore, because these buried residues tend to be highly conserved (they make up the consensus sequence), they blur the resolution for identifying conserved patches on the surface of the protein. Step 5 arranges the LRR amino acid positions into a proximal representation of relative amino acid position, and removal of predicted buried residues in Step 6 is accomplished by deleting the consensus residue columns from further analysis. This generates a representation of the predicted LRR surface.

At this step, in repeats where there are a few additional or fewer residues than the LRR consensus sequence dictates, conservation scores from the adjacent residues are automatically contracted or expanded, respectively, to fill the matrix. It is the responsibility of the user to be aware of occasional large (>4-5 amino acid) intervening “loop-out” regions of non-LRR sequence. These areas generally become apparent to users during intermediate steps of RCM, but if not automatically handled correctly by the program, users must manually edit alignments of these larger intervening sequences from the LRR matrix to ensure that remaining columns of LRR residues remain in proper register relative to the LRR consensus. Users should remain aware of any deleted loop-out regions when viewing final RCM-generated conservation maps.

***Step 7: Calculate regional conservation scores based on conservation of adjacent residues***

To identify the extent to which multiple amino acids within small surface regions of the LRR have been conserved, a sliding window function calculates a regional conservation score for each cell in the matrix based on the conservations scores for that cell and the surrounding 24 cells within a 5x5 window. Computationally, the matrix from Step 6 is used to represent a continuous helix (like the primary amino acid sequence of a three-dimensional LRR), so that there is no discontinuity of the sliding window analysis across the right and left edges of the two-dimensional matrix that is displayed for program users. The default regional conservation scoring is center-weighted so that the individual position conservation score of the central cell counts for 16% of the regional conservation score, while the eight immediately adjacent residue positions account for 8-9% each and the outermost sixteen amino acid positions in the 5x5 window account for 0.5-1.5% each. Alternative sliding window weightings can be investigated by users, if desired, by entering other weightings in the final pages of the online implementation of RCM, or by taking the RCM-generated output MATLAB code for any map and subsequently changing inputs for the CONV2 function in MATLAB. For the first two rows and last two rows of a matrix, 5x5 windows are only partially filled by the LRR matrix and regional conservation scores are adjusted accordingly by duplicating the first and last two rows of the matrix, in reverse order, to allow for sliding window analysis (that is, for a matrix of n rows, the first four rows in the matrix are adjusted to be rows 2, 1, 1, and 2, and the last four rows are rows n-1, n, n, n-1). These extra rows are trimmed away for output.

***Step 8: Generate output image of conservation map.***

The utility of RCM is most strongly realized when the regional conservation scores are viewed as a heat map image (e.g., Fig. 1B, Fig. 2 and other figures). Predicted surface amino acid positions shown in deep red have the highest scores (most conserved) and positions in blue have the lowest scores (most diverged). Unless specified otherwise the color scale is maximized for each individual map, to emphasize contrast. As a companion tool, maps can also be generated from RCM that show the residue conservation scores for the individual amino acid position rather than the regional scores, as shown in lower left of Figure 1B and in Supplemental Figure 2. Additional RCM output includes a text file of the numerical scores, which can be used outside of RCM to generate other representations of the results, for example by using MATLAB.
